# Exosomal and Non-Exosomal Transport of Extra-Cellular microRNAs in Follicular Fluid: Implications for Bovine Oocyte Developmental Competence

**DOI:** 10.1371/journal.pone.0078505

**Published:** 2013-11-04

**Authors:** Md. Mahmodul Hasan Sohel, Michael Hoelker, Sina Seifi Noferesti, Dessie Salilew-Wondim, Ernst Tholen, Christian Looft, Franca Rings, Muhammad Jasim Uddin, Thomas E. Spencer, Karl Schellander, Dawit Tesfaye

**Affiliations:** 1 Department of Animal Breeding and Husbandry, University of Bonn, Bonn, Germany; 2 Department of Animal Sciences, Washington State University, Pullman, Washington, United States of America; Institute of Cancerology Gustave Roussy, France

## Abstract

Cell-cell communication within the follicle involves many signaling molecules, and this process may be mediated by secretion and uptake of exosomes that contain several bioactive molecules including extra-cellular miRNAs. Follicular fluid and cells from individual follicles of cattle were grouped based on Brilliant Cresyl Blue (BCB) staining of the corresponding oocytes. Both Exoquick precipitation and differential ultracentrifugation were used to separate the exosome and non-exosomal fraction of follicular fluid. Following miRNA isolation from both fractions, the human miRCURY LNA™ Universal RT miRNA PCR array system was used to profile miRNA expression. This analysis found that miRNAs were present in both exosomal and non-exosomal fraction of bovine follicular fluid. We found 25 miRNAs differentially expressed (16 up and 9 down) in exosomes and 30 miRNAs differentially expressed (21 up and 9 down) in non-exosomal fraction of follicular fluid in comparison of BCB- versus BCB+ oocyte groups. Expression of selected miRNAs was detected in theca, granulosa and cumulus oocyte complex. To further explore the potential roles of these follicular fluid derived extra-cellular miRNAs, the potential target genes were predicted, and functional annotation and pathway analysis revealed most of these pathways are known regulators of follicular development and oocyte growth. In order to validate exosome mediated cell-cell communication within follicular microenvironment, we demonstrated uptake of exosomes and resulting increase of endogenous miRNA level and subsequent alteration of mRNA levels in follicular cells *in vitro*. This study demonstrates for the first time, the presence of exosome or non-exosome mediated transfer of miRNA in the bovine follicular fluid, and oocyte growth dependent variation in extra-cellular miRNA signatures in the follicular environment.

## Introduction

Bovine follicular development and maturation of oocytes is the result of complex and coordinated processes that involve functional and morphological changes in different types of follicular cells and extensive cell-to-cell communication within the follicular microenvironment [1]. Efficient delivery of factors to and from the oocyte at critical stages of development is essential for coordination of folliculogenesis and triggering of different signaling molecules including Kit, members of TGFB, insulin and WNT signaling family [2], [3], growth factors such as GDF9 and BMP15 [4], and hormonal regulation of FSH, LH [5] that are also crucial for oocyte growth and developmental competence. During *in vitro* maturation and fertilization, a fully grown oocyte has better competency than a growing oocyte. Oocyte developmental competence is defined as the ability of an oocyte to resume meiosis, cleave following fertilization, develop to the blastocyst stage, induce a pregnancy and bring offspring to term with good health [6], [7]. The enzyme glucose-6-phosphate dehydrogenase (G6PD) is minimally active in the fully grown oocytes and present at higher level in growing oocytes. The enzyme G6PD can convert the Brilliant Cresyl Blue (BCB) stain from blue to colorless; thus, growing oocytes will have a colorless cytoplasm while the fully grown ones remained blue. With that BCB staining of COC could be used as a method of screening oocytes for their growth status in many species including cattle [8], [9] and sheep [10]. The development of COC to competent status is taking place in follicular microenvironment in which various signal transductions and molecular interactions are taking place between the surrounding cells mediated by the follicular fluid [11].

Follicular fluid is a product of both the transfer of blood plasma constituents that cross the ‘blood-follicle barrier’ and of the secretory activity of granulosa and thecal cells [12]. It has been recognized as a reservoir of biochemical factors useful as non-invasive predictors of oocyte quality. Follicular fluid provides an important microenvironment for oocyte maturation and contains hormones such as FSH, LH, GH, inhibin, activin, estrogens and androgens, pro-apoptotic factors including TNF and Fas-ligand, proteins, peptides, amino acids, and nucleotides [13]. Follicular fluid is at least partly responsible for subsequent embryo quality and development and has some important oocyte-related functions including maintenance of meiotic arrest [14], protection against proteolysis, extrusion during ovulation [15] and as a buffer against adverse haematic influences [12]. As follicular fluid is derived from plasma and secretions of granulosa and theca cells, it is likely that products within follicular fluid may play a role in follicle growth and oocyte developmental competence.

Exosomes have been postulated to play an important role in cell–cell communication, either by stimulating cells directly by surface expressed ligands or by transferring molecules between them. However, the mode of exosome-cell interaction and the intracellular trafficking pathway of exosomes in their recipient cells remain unclear. Exosomes are small membrane vesicles that are released into the extracellular milieu upon the fusion of multivesicular bodies with the plasma membrane. Unlike other cell-secreted vesicles, exosomes are more homogenous with a size range from 40-100 nm in diameter. Exosomes contain a characteristic composition of proteins, and express cell recognition molecules on their surface that facilitates their selective targeting of and uptake by recipient cells [16]. They are natural carriers of variety of coding and non-coding RNA, including microRNAs (miRNAs) [17], which can be transported over large distances through blood to recipient cells and induce *de novo* transcriptional and translational changes in the target cells [17], [18], [19], [20]. These findings support the idea that exosomes might constitute an exquisite mechanism for local and systemic intercellular transfer not only of proteins but also of genetic information in the form of RNA (mRNA and miRNA). Currently, the role of exosomes, present in bovine follicular fluid, in transporting extra-cellular miRNAs within follicular environment and their contribution to follicular growth and oocyte maturation are unknown.

During the dynamic phase of follicular development and oocyte maturation, miRNAs play an important role by coordinating the expression of genes in a spatial and temporal specific manner [21], [22]. Several studies found that miRNAs are not only present in cells but also in different body fluids including plasma, serum, urine, saliva, milk and semen [23], [24], [25], and those miRNAs are commonly termed as extra-cellular miRNAs or circulating miRNAs. Extra-cellular miRNAs are found to be remarkably stable in plasma despite high RNase activity in extracellular environment [23], suggesting that circulating miRNAs may be protected and bypass the harsh conditions in extracellular environment. The dominant model for extra-cellular miRNA transport and stability is associated with exosomes in different bio-fluids [17], [25], [26]. In addition a significant portion of extra-cellular miRNAs are also associated with non-exosomal structures including Argonaute 2 (AGO2), the effector component of the miRNA-induced silencing complex, that mediates mRNA repression in cells [27]. The discovery of miRNAs in body fluids, such as serum and plasma, and their remarkable stability opens up the possibility of using them as noninvasive biomarkers of disease and therapy response [28]. So far, the presence of these extra-cellular miRNAs and their potential role in follicular microenvironment is not known.

The present study was conducted to investigate: 1) the presence of extra-cellular miRNAs in exosomal and non-exosomal fraction of follicular fluid; and 2) differential expression of extra-cellular miRNAs in follicular fluid derived from growing and fully grown oocyte to postulate their role in oocyte growth. This study revealed the presence of extra-cellular miRNAs in bovine follicular fluid and found that the majority of them are associated with exosomes. Moreover, extracellular miRNAs are also present in non-exosomal part of follicular fluid. By comparing growing vs. fully grown oocytes, we found that both exosomal and non-exosomal fractions of follicular fluid carry a distinct set of differentially regulated extra-cellular miRNAs. Furthermore, exosomes can be taken up by surrounding follicular cells and subsequently increase the level of the endogenous miRNA in follicular cells.

## Materials and Methods

### Collection of follicular fluid and follicular cells

Ovaries were obtained from local abattoir (Bernhard Frenken GmbH Vieh- und Fleischhandle Schlachthof Düren, Paradiesstrasse 19, 52349 Düren, Germany) after official permission by the management to use ovaries for research purpose. Ovaries without any visible active corpus luteum were selected and transported to the lab in 0.9% saline solution at a temperature of 38°C in thermo flask within 2 hours after collection. Following washing with new saline solution, antral follicles of 4-8 mm in diameter were blunt-dissected from the ovaries with scissors and forceps. A caliper was used to measure the diameter of the follicles. Follicular materials from each follicle (n  =  120) were harvested individually by breaking onto the plastic sterile culture dish. The cumulus oocyte complex (COC), retrieved from each follicle, was stained using Brilliant Cresyl Blue (BCB) and grouped as a fully grown oocyte (BCB+) or growing oocyte (BCB-) as described previously [29]. The corresponding follicular fluid, granulosa cells and theca cells were collected and stored separately for further analysis. Follicular fluid, containing granulosa cells, was collected in a sterile microcentrifuge tube by centrifugation at 500×*g* for 3 minutes. While follicular fluid was collected as supernatant, the granulosa cells were collected as pellet from the bottom of the tube. After two additional washes in DPBS (Gibco, life technologies, UK) containing 0.02% polyvinyl alcohol (PVA), granulosa cells were stored at −80°C. The theca cell layer of the follicles was peeled and scraped off with a plastic inoculation loop, washed several times for complete removal of granulosa cells, and stored at −20°C in RNA later (Sigma). Following BCB staining, the COCs were also stored at −80°C separately for further use.

### Isolation of exosomes and non-exosomal structures of follicular fluid

In all cases follicular fluids obtained from the two oocyte competence groups were first subjected to centrifugation at 4000×*g* for 10 min to remove cells and cellular debris. The follicular fluid samples were then filtered through 0.22 µm screen to remove particles whose size is more than 200 nm (apoptotic bubbles and microvesicles portion). After that, samples were subjected to centrifugation at 25,000×*g* for 30 minutes to remove further microparticles, and the microvesicles fraction remained in samples after filtration. All centrifugation steps were performed at 4°C. Cell free follicular fluid samples were used for exosome isolation using two different methods namely: Exoquick exosome precipitation kit (SBI System Biosciences, Inc.) and ultracentrifugation procedure. Exosome isolation using Exoquick exosome precipitation kit was performed according to manufacturer’s instruction. Briefly, Exoquick reagent (120 µl) was added to 500 µl of follicular fluid, incubated 12 h at 4°C, and centrifuged at 1500 × *g* for 30 min to obtain pelleted exosomes. The supernatant (non-exosomal fraction) of the samples were collected without disturbing the exosome pellets, and exosome pellets were resuspended in 200 µl of DPBS. Both the exosomes and exosome-depleted supernatant were further used for total RNA including small RNA fractions and protein isolation to be used for miRNA PCR array analysis and detection of marker proteins, respectively.

To get exosomes for stability test and cellular uptake experiment, exosome isolation from independent follicular fluid samples was performed by ultracentrifugation procedure as previously described by other reports [17], [27]. Briefly, cell-free follicular fluid samples from the above procedures were used for ultracentrifugation at 120,000×*g* for 70 minutes at 4°C in a Beckman SWTi55 rotor. While the supernatant fraction was used for protein isolation, the resulting exosome pellet was resuspended in DPBS and centrifuged again at 120,000×g for 70 min at 4°C. Finally, the resulting pellet was resuspended using DPBS and stored for further use.

### Electron microscopy of exosomes

Morphological evaluation of exosomes isolated using Exoquick kit and ultracentrifugation procedures was performed by visualizing them using electron microscope (Leo 922, Zeiss, Germany). For this, 15 µl drops of isolated exosomes in DPBS were placed on parafilm before the Formvar/carbon-coated grids were placed on top of exosome drops and allowed to stand for 5–10 min to absorb the exosomes. The grids with adherent exosomes were transferred to three 30 µl drops of DPBS for washing and subsequently fixed with 2% paraformaldehyde in DPBS for 7 min. Then the grids were incubated with 25 µl drops of 2% uranyl acetate followed by examination with electron microscope.

### Western blot analysis of proteins from exosomal and non-exosomal fraction

Exosomal proteins were isolated from organic phenol fraction during total RNA isolation using Qiagen miRNeasy mini kit and resuspended in 8M urea. Fiftyµg of protein from each sample were loaded and resolved in 12% SDS-PAGE polyacrylamide gels (Bio-Rad, Corp., Hercules, CA, USA) and then transferred to nitrocellulose membranes (Biotrace NT, Pall life Sciences, Pensacola, FL, USA). Membranes blocked (5% non-fat dried milk in TBST) for 1 h at room temperature, and incubated separately with antibodies raised against CD63 (ExoAB Antibody, SBI, CA, USA), EIF2C2 (0.4 µg/ml; Santa Cruz Biotechnology Inc, USA) and cytochrome C (CYCS; 0.4 µg/ml; Santa Cruz Biotechnology Inc, Texas, USA) overnight at 4°C. After subsequent washing, membranes pre-incubated with the respective primary antibody were further incubated with a horseradish peroxidase conjugated anti-goat, anti-rabbit and anti-mouse secondary antibodies (Santa Cruz Biotechnology Inc., USA), respectively. After additional washes, membranes were incubated for 5 min in chemiluminescent substrate (Thermo Scientific, Waltham, USA), and immunoreactive proteins were visualized with a Chemidoc XRS (Bio-Rad) instrument.

### RNA isolation and reverse transcription

Total RNA (including miRNA) were isolated from exosome preparations and exosome-depleted fraction of follicular fluid using miRNeasy Mini Kit (Qiagen, Hilden, Germany) according to manufacturer’s instructions with some modification adopted for liquid sample. Total RNA (including miRNA) was isolated from theca cells, granulosa cells and cumulus oocyte complexes using the same kit according to manufacturer’s instruction. Total RNA concentration and purity were determined using a NanoDrop ND-1000 spectrophotometer. Moreover, prior to reverse transcription procedure RNA samples from exosomal and non-exosomal fractions were checked for the presence or absence of PCR inhibitors by doing real time quantification of selected miRNAs using cDNA samples generated from different levels of RNA input during cDNA synthesis (0.5 µl, 1 µl, 2 µl and 4 µl of total RNA in 10 µl of reaction volume). All RNA samples showed linear amplification of the candidate miRNAs for different levels of RNA input, which showed the absence of PCR inhibitors and good quality RNA (data not shown). A reverse transcription reaction was performed using the miRCURY LNA™ Universal RT microRNA PCR system (Exiqon, Denmark) according to the manufacturer’s instructions. In brief, approximately a total of 100 ng of total RNA, including small RNA, were anchor-tailed with a poly(A) sequence at their 3'end and then reverse transcribed into cDNA using a universal poly(T) primer with a 3'end degenerate anchor and a 5' end universal tag.

### miRNA profiling and expression analysis

The expression of miRNAs in exosomal and non-exosomal fraction of follicular fluid derived from follicles with growing (BCB-) or fully grown (BCB+) oocyte was performed using microRNA ready-to-use PCR Human Panels (I + II) (Exiqon, Denmark), which contains 748 mature miRNA sequences along with 8 empty wells, 6 intra-plate calibrator and 6 endogenous controls. A SYBR green based real time PCR technology was used for signal detection during quantification. Prior to real time PCR analysis the cDNA products from each samples were diluted 100-fold and mixed with ready to use SYBR-green master mix. Then the cDNA with master mix was robotically pipetted to a 384-well PCR plate containing miRNA specific primers. The real time PCR was run on a ABI-7900HT thermocycler (Applied Biosystems) using the following thermal-cycling parameters: 95°C for 10 min, 40 cycle of 95°C for 10 sec, 60°C for 1 min followed by a melting curve analysis. Raw Ct values were calculated as recommended by Exiqon using the RQ manager software v1.2.1 (Applied Biosystems) with manual settings for threshold and baseline, i.e. all miRCURY assays were analyzed using a ΔRn threshold of 60 and baseline subtraction using cycles 1–14. The PCR data were analyzed using web-based PCR array data analysis software (http://pcrdataanalysis.sabiosciences.com/pcr/arrayanalysis.php). The raw miRNA data was first normalized using a global normalization method. To minimize the potential noise introduced by measurements below detection threshold, miRNAs with Ct value greater than 35 in all groups were considered as undetected. Based on their enrichment in exosomal and non-exosomal fraction of follicular fluid from fully grown (BCB+) or growing (BCB-) oocytes, 8 candidate miRNAs were selected for further characterization of their expression in surrounding follicular cells. For this individual, miRNA specific LNA-TM primer assays were used to investigate their expression in triplicate cDNA samples obtained from the surrounding follicular cells from the contemporary oocyte competence group. For each real time PCR run using sequence specific miRNA primer assays the amplification plot and melting curve are generated to evaluate the PCR amplification specificity and purity. Relative expression of each miRNA was analyzed using a comparative CT (2−ΔΔCT) method (26) and global normalization strategy was employed to normalize the data (27).

### miRNA target prediction

To predict miRNA targets, we used miRecords at http://mirecords.biolead.org/, which contains animal miRNA targets according to combinations of the widely used target prediction programs DIANA MicroTest, Micro Inspector, mirTarget, miRDB, miRanda, TargetScanS, and PicTar and experimentally supported targets from TarBase. For miRNAs annotated with *, their target genes are not present in miRecords database, therefore we used miRDB to predict the target genes list for those miRNAs. The predicted miRNA target genes were analyzed by using the DAVID Bioinformatic Resource (http://david.abcc.ncifcrf.gov/) server for Annotation, Visualization, and Integrated Discovery to identify the pathway distribution. These pathways were presented according to the Kyoto Encyclopedia of Genes and Genomes (KEGG) database (http://www.genome.jp/kegg/).

### Stability of exosomal miRNA under in vitro culture conditions

To check the stability of exosomes under *in vitro* culture condition, follicular fluid samples were collected from slaughterhouse ovaries were used to isolate exosomes by ultracentrifugation according to previously mentioned protocol (see above). After two additional washes in DPBS exosomes were resuspended in 800 µl of F-12 media containing 10% exosomes-free foetal bovine serum (FBS, Invitrogen, South America origin**)**. Exosomes were pre-cleared from the FBS *via* filtration using a 0.22 µm filter (Millipore) and ultracentrifugation at 120,000 *g* for 2 hours. The samples were incubated with different time point at 37°C in humidified atmosphere of 5% CO2 for 0 hr, 6 hrs, 12 hrs and 24 hrs. After incubation total RNA was isolated and individual miRNA expression was measured as described above.

### Exosome labeling

Exosomes isolated from follicular fluid using ultracentrifugation were subjected to fluorescent labeling using PKH67 dye (Sigma-Aldrich), which is a green fluorescent dye that labels the lipid membranes, according to the manufacturer’s instructions. Briefly, exosomes in DPBS were resuspended in 1 ml of diluent C, mixed with freshly prepared PKH67 in diluent C at a final concentration of 5×10^−6^ M, and incubated for 3–5 min. Labeling was stopped by addition of an equal volume of exosome-free FBS and incubate for 1 min, followed by the addition of F-12 media with 10% exosome-free FBS to fill up the centrifuge tube and ultracentrifugation for 30 min at 120,000×*g*. After two additional washes in F12 media (with 10% exosome-free FBS) using ultracentrifugation, the exosomes were resuspended in 100 µl of F12 media with 10% exosome-free FBS.

### Co-incubation of bovine granulosa cells with labeled exosomes

Primary cultures of bovine granulosa cells were established as previously described [30] to undertake labeled exosomes uptake experiment. For this, granulosa cells isolated from bovine follicles were washed once in calcium-magnesium free PBS (CMF) followed by centrifugation at 500X*g* for 5 minutes and resuspended in 2 ml of RBC lysis buffer for 3-5 minutes to remove erythrocytes. Osmality was restored by adding 8 ml of F-12 media containing 10% exosomes free FBS. After two additional washes with media cell viability was determined by a trypan blue exclusion test. Granulosa cells (approximately 45000 viable cells) were seeded on the chamber slide (Lab-Tek™, Thermo Scientific, USA) and cultured for 24 hours in DMEM/F-12 media supplemented with 10% exosomes free FBS, penicillin (100 U/ml) and streptomycin (100 µg/ml) at 37°C in a humidified atmosphere of 5% CO_2_. On the next day, the cells were washed twice with DMEM/F-12 media to remove dead cells and DMEM/F-12 medium containing 10% exosome-free FBS supplemented with labeled exosomes and co-cultured for 24 hrs. After 24 hours of co-culture with labeled exosomes, granulosa cells were washed three times with DPBS and fixed with 4% paraformaldehyde and mounted in mounting medium containing DAPI and observed under a laser scanning confocal microscope (LSM710-Carl Zeiss). These experiments were performed three times, and a parallel negative control was run each time in which cells were co-incubated with sterile PBS instead of exosomes after labeling with fluorescent dye.

### Exosome mediated transmission of miRNAs in granulosa cells *in vitro*

Once the uptake of labeled exosomes by granulosa cells was confirmed, exosomes were isolated from independent follicular fluid from follicle containing BCB+ or BCB- oocytes. After confirming the candidate miRNA enrichment in each group of exosomal samples, a fraction of exosomes was co-incubated with granulosa cells. After 24 hrs of co-culture, the granulosa cells were washed three times in DPBS, collected using 200 µl of trypsin and snap frozen in lysis buffer before using for RNA isolation. The same volume of DPBS added to culture media was used as negative control.

### Quantitative analysis of selected target genes after exosome transfection

To end up with limited number candidate genes for our analysis, we have refined the selection criteria based on the sequence compelementarity between 5′ “seed” region of miRNAs and the 3′-UTR of mRNAs of the potential target genes [31]., As in animals, as little as 6 bp match with the target mRNA can be sufficient to suppress gene expression [32], [33], we searched for target genes with 3′-UTR target sites for a 7-mers seed match (position 2–8) with the seed region of the respective miRNA. Based on this criteria and their potential involvement in critical pathways for follicular development, a total of seven target genes (ITGA3, MAP3K1, SOCS4, BRMS1L, ZNFX1, CD44 and VEGFA) were selected for quantitative real time PCR analysis after exosome mediated transfer of miRNAs in cultured granulosa cells. The respective primers were designed by using FASTA product of the GenBank mRNA sequences for *Bos taurus* using Primer3 program (**Table S6**). Portion of total RNA (used for miRNA abundance study) isolaed from exosome transfected granulosa cells was used for cDNA synthesis performed as described elsewhere [34]. The corresponding cDNA samples were used to quantify all the target genes and GAPDH as endogenous controls [35] in ABI PRISM® 7000 sequence detection system instrument (Applied Biosystems, Foster City, CA, USA). The qRT-PCR was set up using a 2 µl first-strand cDNA template, 7.4 µl deionized H_2_O, 0.3 µM of forward and reverse gene specific primers and 10 µl 1× Power SYBR Green I (Bio-Rad) master mix with ROX as a reference dye. The thermal cycling conditions were 3 min at 95°C followed by 40 cycles of 15 seconds at 95°C and 1 min at 60°C. Relative expression of each mRNA was analyzed using a comparative CT (2^−ΔΔCT^) method.

### Statistical analysis

All graphs were made and statistical analyses performed using GraphPad Prism, or Microsoft Excel 2010. All experiments were performed a minimum of three times. When two groups were compared (i.e., BCB- vs. BCB+) a Student’s *t*-test was used to detect differences between treatment groups. A *P value of* ≤ 0.05 was considered to be significant. Data are expressed as mean ± SEM of replicates.

## Results

### Efficient recovery of exosomes from bovine follicular fluid and visualization

**Figure 1:**
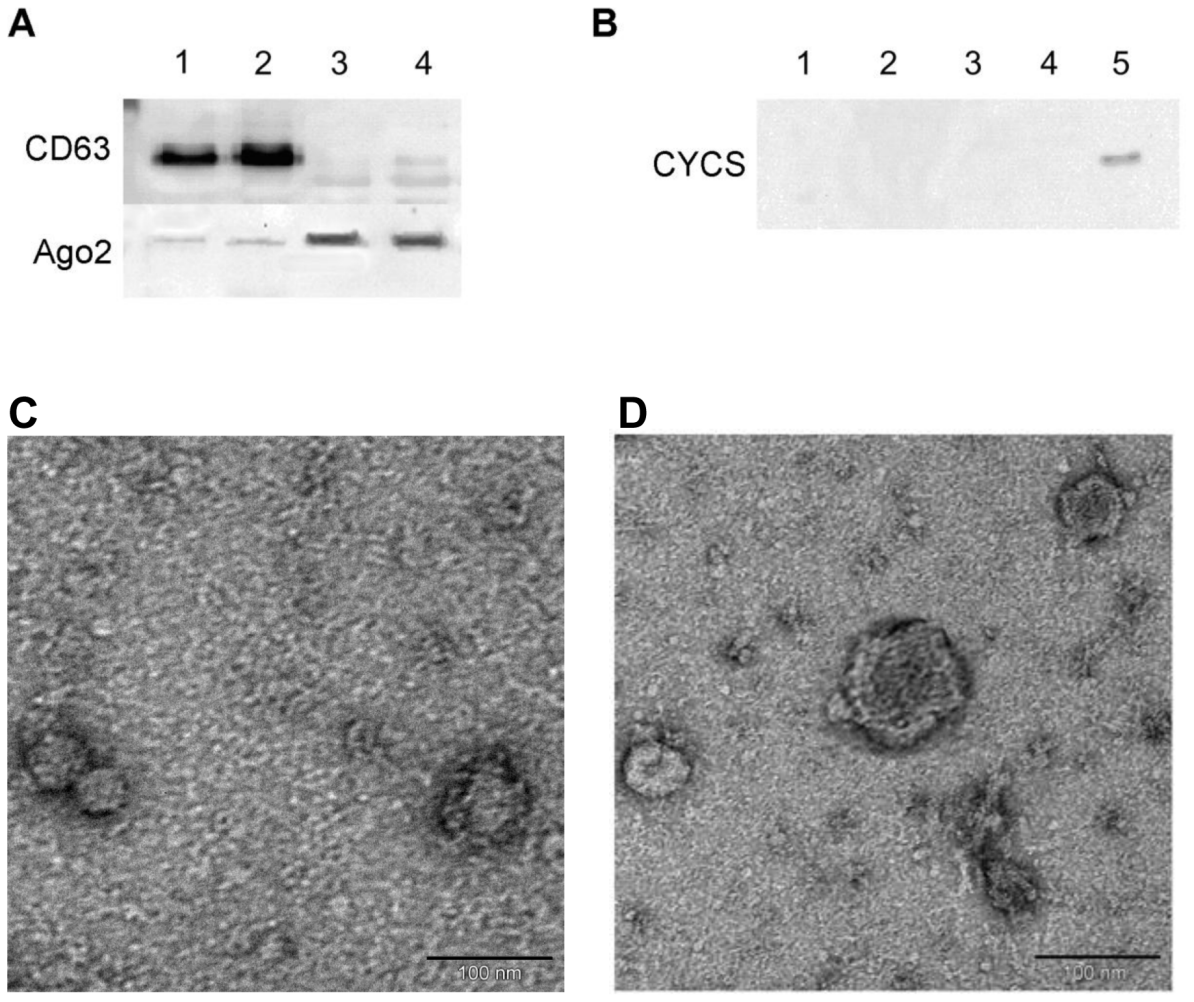
Western blot analysis (A & B) of exosomal and non-exosomal fractions of follicular fluid and morphological presentation of exosomes using electron microscopy (C & D). (A) Proteins (50 µg) from exosomal fraction (lane 1 & 2) and non-exosomal fraction (lane 3 & 4) of follicular fluid isolated either by ultracentrifugation (lane 1 & 3) or Exoquick kit (lane 2 & 4) were resolved on a 12% SDS-PAGE gel to detect CD63 and Ago2 proteins. (B) Confirmation of absence of detection of CYCS (mitochondrial protein) in both exosomal and non-exosomal proteins as isolated by ultracentrifugation (lane 1 & 3) and Exoquick kit (lane 2 & 4). A known cellular origin protein was used as a positive control (lane 5). Morphological characterization of exosomes isolated from bovine follicular fluid using Exoquick kit (C) and ultracentrifugation (D) by transmission electron microscopy. The scale bar indicates 100 nm.

Exosomes were isolated from follicular fluid using two different methods, namely exoquick precipitation and differential ultracentrifugation. The specificity of those isolation procedures were characterized at the protein level by the presence of CD63, a membrane protein, for exosomes, Ago2 protein in the non-exosomal fraction of the follicular fluid, and the absence of cellular cytochrome C (CYCS) for both fractions. As shown in **Figure 1A**, CD63 protein was found in both exosomes isolated by ultracentrifugation (lane 1) or Exoquick precipitation (lane 2). Efficient recovery of exosomes by both methods was confirmed by absence of Ago2 protein contamination. The non-exosomal fraction of follicular fluid from the Exoquick isolations contained an ignorable amount of CD63 protein (**Figure 1A** **lane 4**); however, both non-exosomal fractions (either from exoquick preparation or ultracentrifugation) contained Ago2 protein (**Figure 1A** **lanes 3 and 4**). Furthermore, CYCS (mitochondrial protein) was not present in either the exosomal or non-exosomal fraction of follicular fluid isolated by both methods, indicating that those protein samples are free of any protein of cellular origin (**Figure 1B**). To investigate the morphological characteristics of exosomes in follicular fluid, exosomes were isolated using Exoquick Kit and differential ultracentrifugation. The exosomes were absorbed on formvar carbon coated grids, fixed with PFA, contrasted using uranyl acetate and observe under electron microscope. Observation by electron microscope revealed that both preparations contained vesicles (**Figure 1C & D**) which were similar in size (40-100 nm) to the previously described exosomes [**17]**, [36].

### Detection of miRNAs in exosomal and non-exosomal fraction of bovine follicular fluid

The expression of mature miRNAs were examined in exosomal and non-exosomal fractions isolated from follicular fluid of each catagory (BCB+ or BCB-) using microRNA ready-to-use PCR Human Panels (I + II) (Exiqon, Denmark). After comparative analysis of these mature human miRNA sequences to the recent bovine mature miRNA sequences available in miRBase version 19 (http://www.mirbase.org/), we found 241 miRNAs to be completely identical with bovine sequence, 126 miRNAs showed differences in size due to addition or deletion of up to 5 nucleotides, 58 miRNA sequences contained mismatches within the mature sequences, out of which 27 miRNAs showed only single mismatch. But no bovine homologous sequences were found for the 323 miRNAs in the present miRBase databank. The presence of extra-cellular miRNAs in exosomal and non-exosomal fractions of bovine follicular fluid obtained from both follicular categories were determined by Ct value i.e. the number of cycles required to get a fluorescence signal above background. Those miRNAs were considered as detected when their Ct value < 35, while those with Ct value of ≥ 35 were considered as non-detected. This threshold level is set based on the previous experiences in detection of transcripts and confirmation of products on agarose gel in our lab. Moreover, similar studies on detection of RNA molecules in plasma samples also use the same threshold level to determine the detection level of transcripts [37], even as high as ≤37 cycle as a threshold to determine the level of detection [20]. This analysis revealed that both exosomal and non-exosomal fractions of follicular fluid contain a large array of miRNAs (detected miRNAs were listed in **Table S1**). Among the 748 miRNAs profiled, 509 and 356 miRNAs were detected in exosomal and non-exosomal fraction, respectively. From the detected miRNAs, a total of 331 miRNAs were commonly detected in both exosomal and non-exosomal fractions of follicular fluid. However, 178 and 25 miRNAs were detected only in exosomal and non-exosomal fractions, respectively (**Figure S1**).

### Differential expression of miRNAs in exosomal and non-exosomal fraction of follicular fluid based on oocyte competence

We next tested our hypothesis that miRNAs are present as extra-cellular molecules in follicular fluid and have different expression patterns depending on the stage of oocyte growth or competency. The miRNAs were profiled in both exosomes and non-exosomal fraction of follicular fluid of BCB+ & BCB- oocyte origin. Since global normalization showed better results within groups compared to spiked-in miRNA or mammalian U6 [37], we used the same normalization procedure (correction with the sum of the expression levels of detected miRNAs) to normalize the expression in real time PCR data analysis. Comparisons were made for expression analysis of miRNA in follicular fluids of follicles containing growing oocyte (BCB-) versus a fully grown oocyte (BCB+) in exosomal and non-exosomal fraction separately. The number of differentially expressed miRNAs (P<0.05) by fold change (1.5- and 2-fold) for each comparison is summarized in **Table 1**. Accordingly, 25 and 30 miRNAs in the exosomal and non-exosomal fraction, respectively, were differentially regulated (≥2 fold change, P< 0.05) between follicular fluids from BCB+ & BCB- groups (**Table 2**).

**Table 1:** Summary of the number of differentially expressed miRNAs obtained from comparisons (growing vs. fully grown) in exosomal and non-exosomal fraction of follicular fluid.

	Growing vs. Fully grown oocyte groups		
	Exosomal	Non-exosomal	Overlap
Number of DE miRNAs (P<0.05)	40 (↑23, ↓17)	41 (↑30, ↓11)	1
Number of DE miRNAs (P<0.05 & fold change > 1.5)	38 (↑22, ↓16)	35 (↑24, ↓11)	1
Number of DE miRNAs (P<0.05 & fold change > 2)	25 (↑16, ↓9)	30 (↑21, ↓9)	0

DE: differentially expressed.

↑: Up-regulated in the growing vs. fully grown comparison.

↓: Down-regulated in the growing vs. fully grown comparison.

**Table 2:** List of differentially expressed microRNAs in exosomal and non-exosomal fraction of bovine follicular fluid obtained from growing vs. fully grown oocyte comparison.

Exosomal Portion (Growing vs. fully grown)			Non-exosomal Portion (Growing vs. fully grown)		
MicroRNA Name	Fold change (BCB-/BCB+)	P value	MicroRNA Name	Fold change (BCB-/BCB+)	P value
miR-654-5p	49.64	0.0152	miR-19b-1*	9.54	0.0098
miR-640	8.09	0.0408	miR-29c	7.8	0.0138
miR-582-5p	5.02	0.0120	miR-659	7.72	0.0018
miR-449b	4.39	0.0120	miR-29a	7.51	0.0000
miR-155	3.36	0.0021	miR-424*	6.49	0.0470
miR-573	2.95	0.0005	miR-133a	6.07	0.0417
miR-451	2.78	0.0468	miR-193a-3p	5.64	0.0152
miR-221	2.76	0.0001	miR-617	5.62	0.0489
miR-363	2.73	0.0441	miR-145*	4.83	0.0313
miR-199a-5p	2.4	0.0491	miR-423-5p	4.19	0.0367
hsa-let-7c	2.35	0.0454	miR-365	3.7	0.0419
miR-491-5p	2.26	0.0353	miR-99a*	3.59	0.0266
miR-21	2.23	0.0282	miR-505	3.42	0.0048
miR-132	2.17	0.0349	miR-101*	3.12	0.0463
miR-873	2.16	0.0357	miR-15a	2.8	0.0309
miR-324-3p	2.01	0.0219	miR-222	2.72	0.0425
miR-450b-3p	–2	0.0002	miR-103	2.67	0.0108
miR-191*	–2.05	0.0015	miR-654-3p	2.64	0.0137
miR-26b*	–2.06	0.0013	miR-532-5p	2.45	0.0339
miR-1272	–2.27	0.0474	miR-145	2.18	0.0088
miR-29a*	–2.3	0.0080	miR-574-3p	2.03	0.0118
miR-30b	–2.44	0.0375	miR-184	–2.8	0.0119
miR-33a*	–2.59	0.0400	miR-425*	–2.99	0.0295
miR-526b*	–35.23	0.0399	miR-186	–3.43	0.0143
miR-373	–265.11	0.0210	miR-519d	–3.99	0.0211
			miR-302c	–5.02	0.0052
			miR-934	–7.75	0.0297
			miR-30e*	–9.28	0.0119
			miR-18a*	–14.35	0.0106
			miR-381	–17.41	0.0478

(P<0.05) with at least two-fold change.

Of the 25 exosomal miRNAs that were differentially expressed, 16 and 9 miRNAs were up- and down-regulated, respectively in the growing oocyte (BCB-) group. Among the up-regulated miRNAs, miR-654-5p & miR-640, and down-regulated miRNAs, miR-373 & miR-526b*, displayed the greatest fold change difference in the exosomal fraction of follicular fluid. Among the 30 non-exosomal differentially expressed miRNAs, 21 and 9 miRNAs were found to be up- and down-regulated, respectively, in the growing oocyte group (BCB-). However, from the up-regulated miRNAs, miR-19b-1* and miR-29c, and from down-regulated miRNAs, miR-381 and miR-30e*, had the highest fold change in the non-exosomal fraction of follicular fluid. Importantly, there was no overlap observed between the differentially expressed miRNAs identified from exosomal and non-exosomal fraction, suggesting that each fraction of follicular fluid transport a distinct subset of differentially regulated miRNAs.

### Target prediction, biological functions and canonical pathways identified for differentially expressed miRNAs

**Figure 2:**
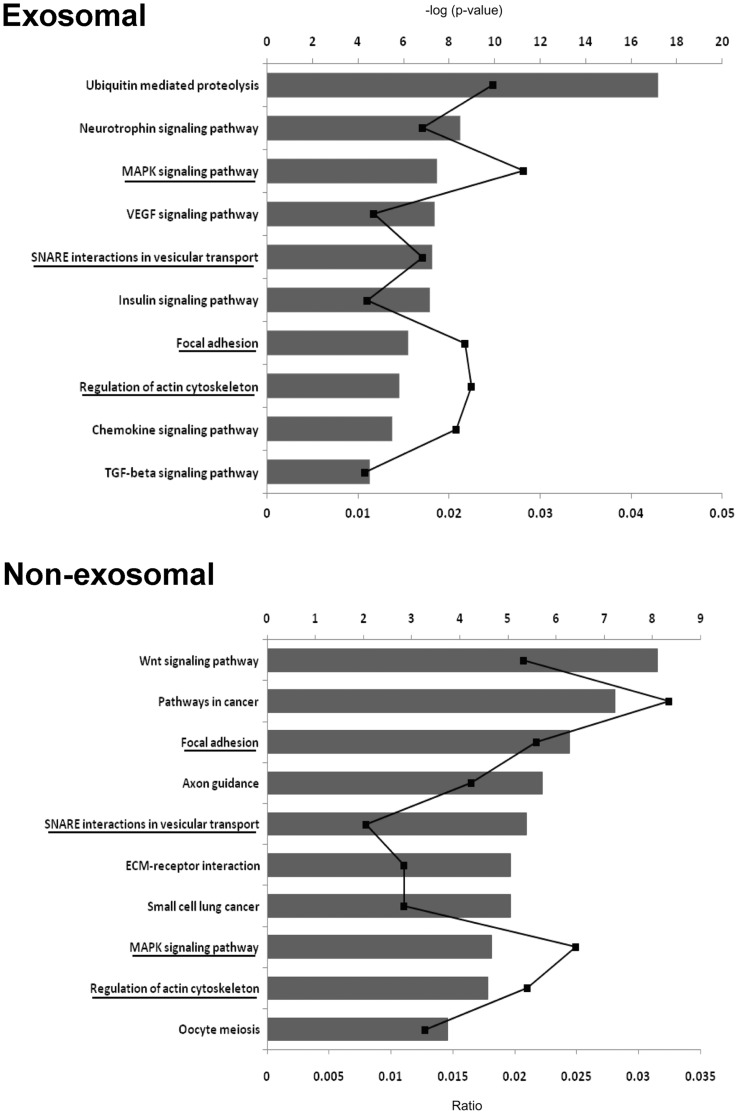
Enriched pathways predicted to be targeted by differentially expressed miRNAs in both fraction of follicular fluid. Pathways enriched by genes predicted to be targeted by miRNAs highly abundant in exosomal and non-exosomal fraction of follicular fluid derived from follicles with growing oocytes (BCB-) compared to fully grown oocytes (BCB+) group. The bar graphs represent the –log(p-value) for each pathway, while the line diagram indicates the ratio of number of genes from the data set to the total number of genes involved in respective pathways. The common pathways for differentially expressed miRNAs in exosomal and non-exosomal fraction of follicular fluid are underlined.

To understand the biological relevance of the miRNA signature in follicular fluid (both exosomal and non-exosomal fraction) we have performed *in silico* analysis in order to identify the potential target genes of the differentially expressed (≥2-fold change, P<0.05) up-regulated miRNAs of growing oocyte (BCB-) group. As the number of experimentally validated miRNA targets is limited, we used miRecords at http://mirecords.biolead.org/, which contains animal miRNA potential targets according to combinations of the widely used target prediction programs DIANA, MicroTest, Micro Inspector, mirTarget, miRDB, miRanda, TargetScanS, and PicTar. We considered a gene as a potential target of a specific miRNA when it was predicted by at least 4 target prediction programs. A total of 7,960 and 4,948 potential targets were identified through this process for differentially expressed exosomal and non-exosomal miRNAs respectively. The top 6 predicted target genes for each of differentially expressed miRNAs are listed in **Table S2**. We then used DAVID (http://david.abcc.ncifcrf.gov/) server to identify the gene ontology (GO) and significantly enriched canonical pathways (P<0.01) of all the predicted targets. As shown in **Figure 2**, although there is no overlap at the differentially expressed individual miRNA level, there is a much higher degree of convergence at pathway levels regulated by differentially expressed miRNAs.

Our analysis for GO revealed that the genes targeted by up-regulated miRNAs in both exosomal and non-exosomal fraction of follicular fluids of the growing oocyte group were associated with transcriptional categories (such as transcription, regulation of transcription, transcription factor activity), transport, secretion and gene expression (**Table S3**). As shown in **Figure 2**, ubiquitin-mediated proteolysis, neurotrophin signaling pathway, and MAPK signaling pathway are the most enriched pathways for the target genes of differentially expressed exosomal miRNAs. However, WNT signaling pathway, pathways in cancer and focal adhesion are the most enriched pathways for the genes potentially targeted by non-exosomal miRNAs. Furthermore, pathways involved in various signal transduction and cell-cell interactions such as VEGF signaling pathway, insulin signaling pathway, focal adhesion, pathways in cancer, and oocyte meiosis TGF-beta signaling pathways are also significantly enriched in both exosomal and non-exosomal fractions of follicular fluid from follicles containing growing oocytes. For detail information about the pathways please see **Table S4 and Table S5.**

### Expression analysis of candidate miRNAs in surrounding follicular cells

**Figure 3:**
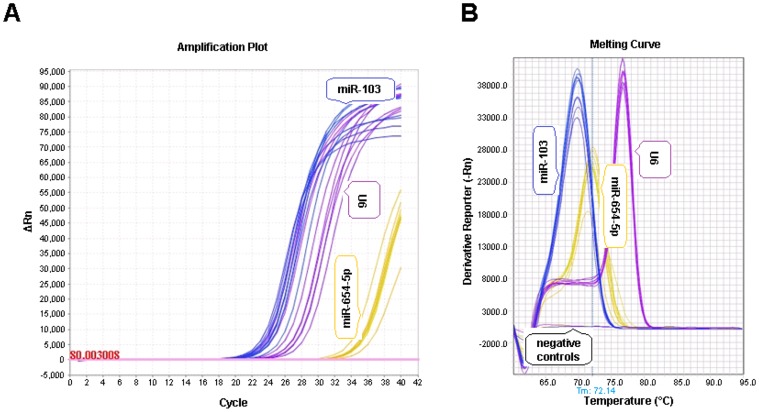
Representative amplification plot (A) and melting curve (B) of miR-654-5p, miR-103 and U6. The amplification plot and melting curve was generated during analysis og the expression of miR-654-5p and the two endogenous controls (miR-103 and U6) in granulosa cells co-cultured with exosomes derived from follicular fluid containing growing (BCB-) oocytes. The amplification plot shows the rise in fluorescence signal above the threshold level (with ▵Rn  =  80.00) for the respective miRNA assays during the PCR run. The signal from the negative controls (RNase-free water) remained below the threshold line. The melting curve generated at the end of each PCR run showed the specificity of amplification, which is evidenced by a single peak for each miRNA assay. The negative controls showed no peaks and the signal remained horizontal.

**Figure 4:**
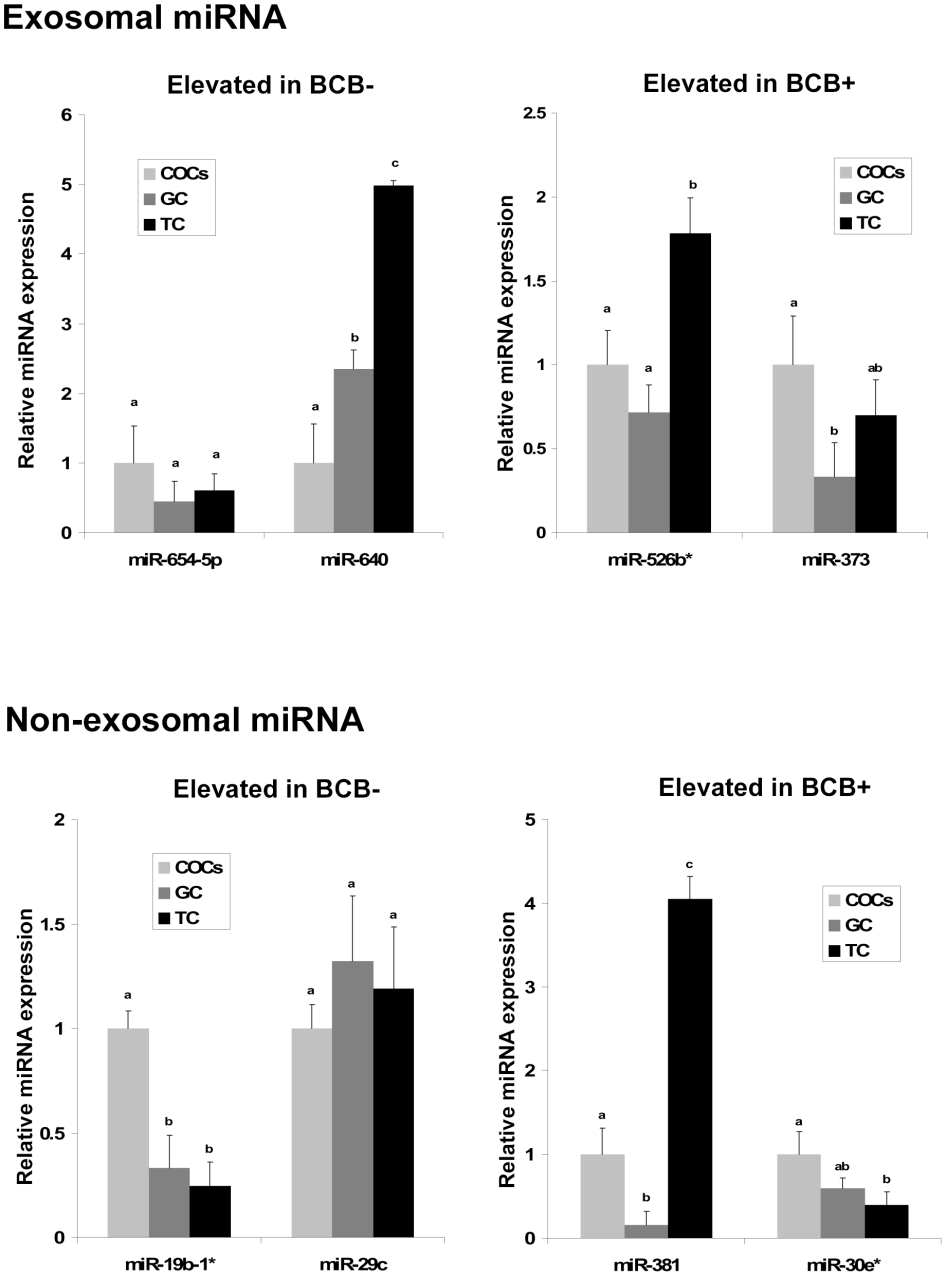
Analysis of exosomal and non-exosomal miRNAs in follicular cells. Expression patterns of miR-654-5p and miR-640 (up-regulated in exosomal fraction follicular fluid derived from follicles containing BCB- oocytes) and miR-526b* and miR-373 (up-regulated in exosomal fraction of follicular fluid from BCB+ groups) were investigated in surrounding follicular cells namely: cumulus oocyte complex (COCs), granulosa cells (GC), and theca cells (TC) from the same category of follicle which were used for miRNA PCR array analysis. Four miRNAs namely: miR-19b-1*& miR-29c (enriched in non exosomal fraction of follicular fluid from BCB-) and miR-381 & miR-30e* (enriched in non exosomal fraction of follicular fluid from BCB+ group) were also investigated for their expression in surrounding follicular cells. The data is presented as relative abundance of different miRNAs in different cell types compared to their expression in COCs as a control. Error bars represents means ± SD of three biological replicates and different superscript letters (a-c) denote a significant difference between groups (P<0.05) as determined by Student’s *t* test.

Based on their expression profile in both exosomal and non-exosomal fraction of the follicular fluid containing either growing or fully grown oocytes, we selected 8 candidate miRNAs to investigate their expression in the surrounding follicular theca cells (TC), granulosa cells (GC) and cumulus oocyte complex (COCs) using individual PCR assays. During each single miRNA PCR assay run the amplification plot and melting curve were generated to observe the detection intensity and the specificity of amplification, respectively, in all samples under investigation. Representative amplification plot and the corresponding melting curve for candidate miRNA and endogenous controls are illustrated in **Figure 3**. The PCR array results revealed that, in follicular fluid from follicles containing growing oocytes (BCB-), miR-654-5p and miR-640 (in exosomal) and miR-19b-1* and miR-29c (in non exosomal) were highly abundant (**Table 2**). Similarly, miR-526b* and miR-373 (in exosomal) and miR-381 and miR-30e* (in non-exosomal) were more abundant in follicular fluid from follicles with a fully grown oocyte (BCB+) (**Table 2**). Comparative expression analysis of 8 candidate miRNAs in follicular cells showed that they are present in all cell types with different expression level. While exosomal miRNAs namely, miR-640 and miR-526b* were more abundant in theca cells, miR-373 was detected at higher level in COCs. However, no significant difference was observed in the expression level of miR-654-5p across different cell types (upper panel of **Figure 4**). Similar analysis for non-exosomal miRNAs shows that miR-19b-1* and miR-30e* were highly abundant in cumulus oocyte complex (COCs) whereas miR-381 was highly abundant in theca cells. However, no significant difference was observed for miR-29c across the cell types (lower panel of **Figure 4**).

### Stability of exosomal miRNAs under culture conditions

**Figure 5:**
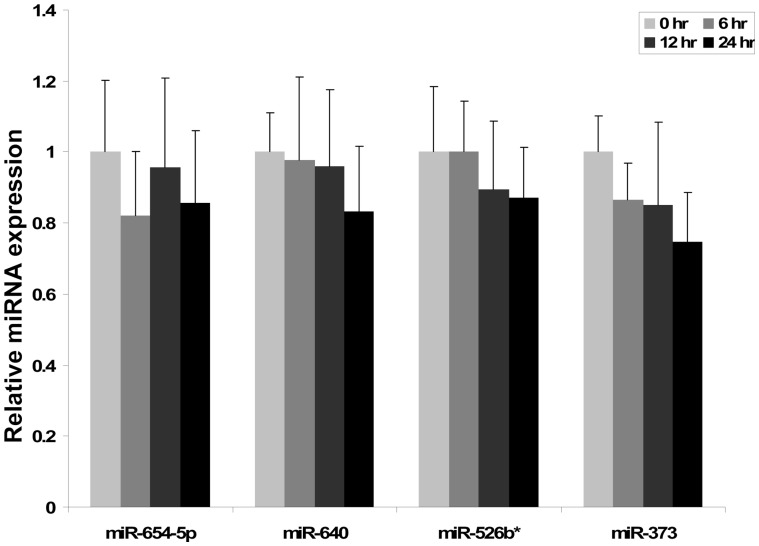
Stability of exosomal miRNAs under *in vitro* culture conditions. Exosomes, isolated using differential ultracentrifugation from follicular fluid, were incubated under *in vitro* cell culture conditions (37°C & 5% CO_2_) for 6 hr, 12 hr and 24 hr in a exosome-free culture medium in order to determine the stability of exosomal miRNAs by quantitative real time PCR. Non-cultured exosomes (0 hr) were used as reference controls to check the stability of exosomes coupled miRNAs in different time point. The data is presented as means and SD of three biological replicates.

To demonstrate the stability of exosomes under culture condition (F-12 media + 10% exosomes free FBS, 37°C and 5% CO_2_) we investigate the expression of four candidate exosomal miRNAs at three different time points (6 hr, 12 hr and 24 hr) and compared with freshly isolated exosomes (0 hr) samples derived from follicular fluid irrespective of oocyte competence. We choose miR-654-5p, miR-640, miR-526b* and miR-373 because these miRNAs were differentially expressed in exosomal fraction of follicular fluid and they were also substantially expressed in exosomes. We observed that these exosomal miRNAs of bovine follicular fluid shows more or less stable expression under culture conditions at different time points (**Figure 5**). The *in vitro* culture temperature is similar to body temperature of bovine, thus exosomal miRNAs are stable in follicular fluid (up to 48 hrs, data not presented).

### Exosomes can be taken up by follicular cells and increase endogenous miRNA abundance

**Figure 6:**
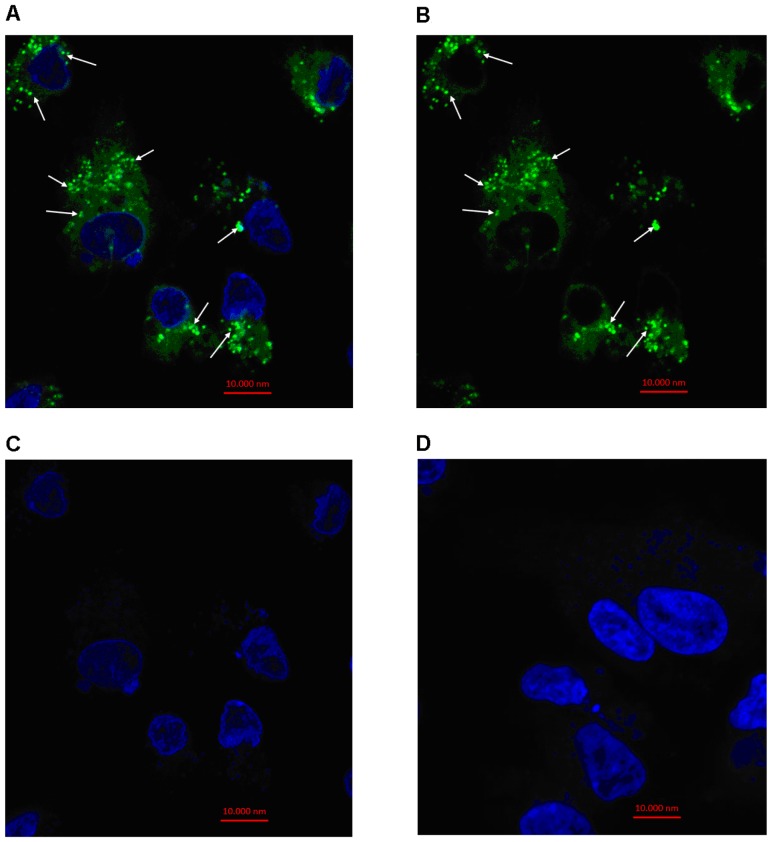
Uptake of PKH67-labeled exosomes by bovine granulosa cells *in vitro*. Exosomes, purified from follicular fluid, were labeled with PKH67 dye and added to primary culture of granulosa cells. Granulosa cells were co-cultured with labeled exosomes isolated from follicular fluid in exosome-free medium for 22 hours under optimum cell culture conditions (37°C & 5% CO_2_). Nuclei are stained blue (DAPI) while PKH67-labeled exosomes are stained green (**Figures 7** **A, B & C**). Arrows indicate exosomes that were taken up by granulosa cells. Granulosa cells cultured in exosomes free medium containing PKH67-labeled sterile PBS served as a negative control (**Figure 7** **D**). Scale bar: 10,000 nm.

To evaluate the potential of exosome mediated transfer of molecules from follicular fluid to the surrounding cells, exosome uptake experiment was conducted using cultured granulosa cells. For this 50,000 viable granulosa cells were seeded to each well of 8-well chamber slide. Purified exosomes from follicular fluid of either BCB+ or BCB- oocyte groups were labeled with a green fluorescent PKH67 dye and added to the culture media of granulosa cell. Following co-incubation period of 24 hr, labeled exosomes treated granulosa cells were washed and fixed and examined by a confocal microscope. Microscopic results showed effective uptake of PKH67 labeled exosomes by cultured bovine granulosa cells (**Figures 6A, B & C**), while granulosa cells cultured with only in sterile PBS suspended PKH67 shows no signals for green fluorescence (**Figure 6D**).

**Figure 7:**
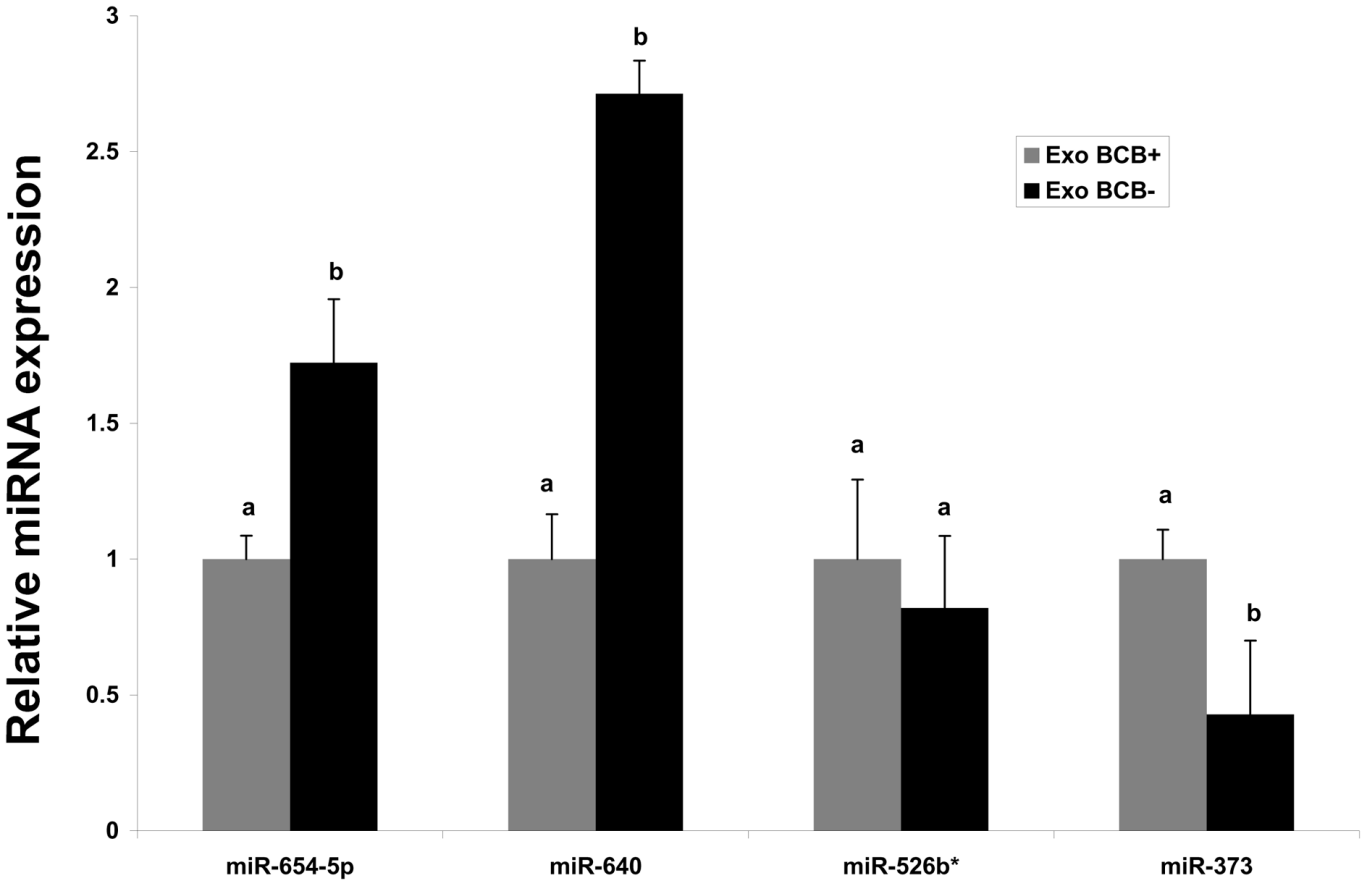
Validation of the enrichment of candidate miRNAs in exosomes taken up by granulosa cells. Purified exosomes from follicular fluid of follicles with growing (BCB-) or fully grown (BCB+) oocytes were subjected to total RNA extraction. Then the expression level miR-654-5p and miR-640 (enriched in exosomes derived from BCB- follicular fluid) and miR-526b* and miR-373 (enriched in follicular fluid derived from follicles with BCB+ oocyte) were investigated by real time PCR. Different superscript letters (a,b) denote a significant difference between groups, such that groups not sharing a similar letter are significantly different form each other (P<0.05). The data is presented as means ± s.d. of three biological replicates.

**Figure 8:**
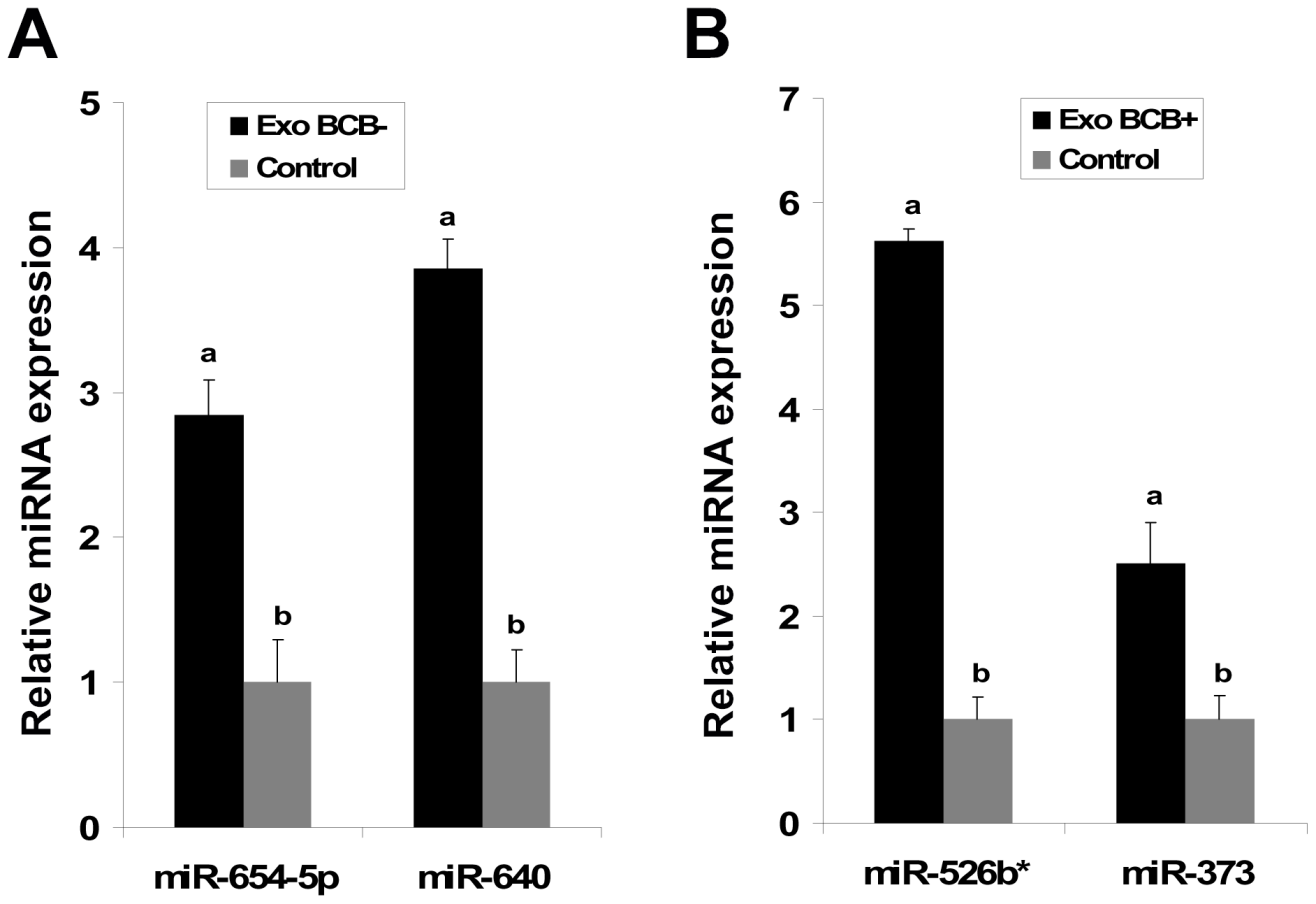
Exosome mediated delivery of miRNAs in bovine granulosa cells *in vitro*. Purified exosomes from follicular fluid of follicles from growing (Exo BCB-) or fully grown (Exo BCB+) oocytes were co-cultured with bovine granulosa cell. After 24 hrs of incubation at 37°C in a humidified incubator, cells were collected and subjected to total RNA extraction. The expression levels of candidate miRNAs were investigated by real time PCR. In both cases the level of endogenous miRNAs were significantly increase compared to untreated controls. Bars with different superscript letters (a,b) are significantly different (P<0.05) from each other. The data is presented as means ± SD of three biological replicates.

To determine whether exosomes mediated transfection of miRNAs could increase the endogenous level of miRNA, we first purified exosomes from follicular fluid of BCB+ or BCB- oocyte source and take fraction of it to check the abundance of candidate miRNAs in those exosome samples. Importantly, we found that miR-654-5p, miR-640 and miR-526b*, and miR-373 were more abundant in exosomes from follicular fluid of follicles containing BCB- and BCB+ oocytes, respectively (**Figure 7**). Then the rest portion of exosomes from BCB+ and BCB- follicular fluid categories were co-cultured separately with primary granulosa cells *in vitro* for 24 hours, after which the expression level of those miRNAs was investigated. Granulosa cells co-cultured with the same volume of sterile PBS were used as negative control. The results revealed that the level of endogenous miRNAs in exosome co-cultured granulosa cells was significantly increased compared to controls (**Figures 8A & B**).

### Changes in expression of target genes following treatment of exosomes in granulosa cells

**Figure 9:**
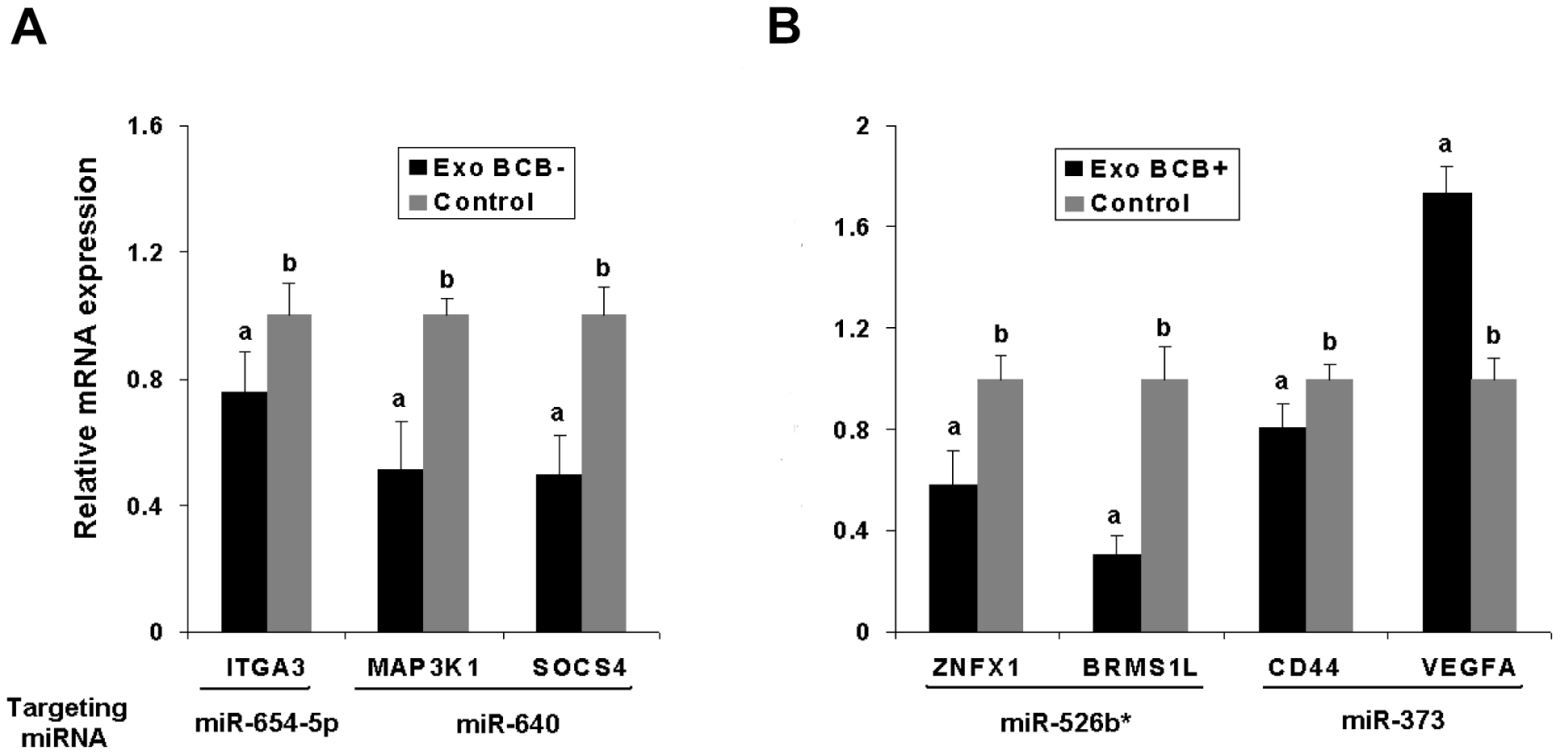
Changes in expression of target genes following exosomes transfection. Quantitative real-time PCR were carried out using gene-selective primers. miRNA target genes were selected using seed match and their involvement in important pathways. To elucidate the exosome mediated uptake of extra-cellular miRNAs can alter the abundance of target mRNAs we used the same exosome co-cultured granulosa cells which was used for miRNA abundance study. The histogram in this figure indicated that significantly lower expression of target mRNAs except VEGFA, suggesting possible involvement of transcriptional regulation of circulating miRNA. Different superscript letters (a,b) denote a significant difference between groups, such that groups not sharing a similar letter are significantly different from each other (P<0.05). The data is presented as means ± SD of three biological replicates.

To determine whether exosome-mediated delivery of miRNA can alter the mRNA abundance of selected target genes, we examined the expression of seven transcripts in granulosa cells collected after treatment with exosomes enriched by candidate miRNAs compared to untreated controls. Based on the criteria mentioned in material and methods part we found ITGA3 (as target of miR-654-5p), SOCS4 & MAP3K1 (as target of miR-640), BRMS1L & ZNFX1 (as target of miR-526b*) and CD44 & VEGFA (as target of miR-373). As shown in **Figure 9**, treatment of granulosa cells with exosomes enriched with candidate miRNAs down-regulated ITGA3, SOCS4, MAP3K1, BRMS1L, YNFX1 and CD44 genes by 20-65% compared to the untreated controls. However, the relative abundance of VEGFA has increased in granulosa cells transfected with exosomes compared with untreated controls (**Figure 9B**).

## Discussion

Recently miRNAs have been detected in extracellular environment mainly in different bio-fluids and their spectra could reflect altered physiological and pathological conditions [17], [25], [38], [39], [40], [41]. To our knowledge, this study is the first report on the presence of extra-cellular miRNAs in bovine follicular fluid, which may differ in its composition depending on the growth status of the oocytes. The results of the present study clearly support our hypothesis that miRNAs are also present in bovine follicular fluid being associated with either exosomes or non-exosomal structures and their signature may represent the altered physiological conditions in the follicular microenvironment.

Various studies have demonstrated that circulating miRNAs are coupled with either exosomes [18], [36] or AGO2 protein complex [27] are able to bypass the high RNases activity in blood stream. By using a systemic approach, we have revealed that there are at least two populations of extra-cellular miRNAs in follicular fluid, namely exosomes and non-exosome associated, in which the former represent the majority of circulating miRNAs in follicular fluid. The specificity of exosome isolation was confirmed by the presence of CD63 in isolated exosomes [17], [42], [43]. To support the hypothesis that along with exosomes AGO2 protein complex may also carry a sub set of miRNA in follicular fluid, we checked the presence of AGO2 protein complex in the non-exosomal fraction of follicular fluid. Furthermore, both exosomal and non-exosomal protein preparations were negative for CYC, which is exclusively expressed in cytosol/mitochondria [20], [44], confirmed the absence of cellular fraction.

In addition to their association with microvesicles/exosomes [18], [39] and non-vesiclar molecules (Ago2 complex associated) [27] componants, very recent report showed that miRNAs may present in blood plasma in association of High Density Lipoprotein (HDL) and delivered them to recipient cells with functional capabilities [45]. In the present study, in addition to the exosomal fraction, we demonstrated that miRNAs were also coupled with non-exosomal fraction (supernatant) of the follicular fluid. Subsequently, we have confirmed the presence of miRNAs in both exosomal and non-exosomal fraction of the follicular fluid using the Human miRNome PCR array platform. Despite using a heterologous approach, the quantitative real time PCR analysis shows detection of the majority of miRNAs in both fractions of follicular fluid indicating the cross species conservation feature of miRNAs between human and bovine as it has been observed in wide range of species [46]. Of the total of 750 miRNAs in the PCR array panel, a total of 509 and 356 miRNAs were detected in exosomal and non-exosomal fraction of follicular fluid respectively (**Figure S1**). Among the detected miRNAs, 331 were commonly found in both fractions, while 178 and 25 miRNAs were detected only in the exosomal and non-exosomal fraction of follicular fluid, respectively. This shows exosome mediated transport of miRNAs is the dominant pathway in bovine follicular fluid compared to the non-exosomal way (Ago2 or HDL) as observed previously in blood plasma and saliva [43]. To explore the possible association of exosomal and non-exosomal miRNA expression with oocyte growth in the follicle, we examined the relative miRNA expression level in exosomal and non-exosomal fractions of bovine follicular fluid collected from follicles containing growing (BCB-) and fully grown (BCB+) oocyte. Subsequently we found 25 and 32 miRNAs to be differentially regulated in exosomal and non-exosomal fraction, respectively between two oocyte groups (BCB- vs. BCB+). The higher number of up-regulated miRNAs in both exosomal (16 miRNAs) and non-exosomal (21 miRNAs) fraction of the follicular fluid of growing oocyte group may indicate the presence of a higher degree of transcriptional activity during the growth phase of the oocyte. In order to evaluate the potential role of these differentially expressed miRNAs, the target genes were predicted bioinformatically and their biological function and gene ontology was determined. The most dominant categories enriched by predicted genes are related to transcription and transport which may indicate that, growing oocytes have higher degree of transcription and translation resulting in efficient RNA management and storage in oocytes as maternal resource [29], [47]. Furthermore, the most significant pathways enriched by predicted targets for up-regulated exosomal miRNAs include ubiquitin-mediated pathway, neurotrophin signaling, MAPK signaling and insulin signaling pathways. All these pathways are known to be involved in ovarian follicular growth and many developmental processes. The ubiquitin mediated pathway is known to modulate oocyte meiotic maturation [48], early mitotic division in developing embryos [49] and plays important role in many cellular processes [50]. While neurotrophin signaling pathway is reported to be important in regulation of oogenesis and follicle formation [51], the MAPK signaling mediates LH-induced oocyte maturation and its activation in cumulus cells is appears to require the permissive effect of the oocyte itself [52]. Similarly, the overrepresented pathways in non-exosomal fraction were WNT signaling pathway and pathways in cancer and focal adhesion. WNT molecules are glycoproteins involved in fetal ovarian development and adult ovarian function including follicular growth, oocyte growth or maturation, steroidogenesis, ovulation and luteinization [2], [53], [54]. Moreover, the higher number of common pathways in both exosomal and non-exosomal fraction may indicate that, miRNAs associated with either exosomes or Ago2 has a complementary function in follicular microenvironment.

To validate the hypothesis that extra-cellular miRNAs in follicular fluid originate from different cell types within follicular microenvironment we have investigated the expression of 8 selected candidate miRNAs in granulosa cells (GCs), theca cells (TCs) and cumulus oocyte complex (COCs). The results revealed that all these miRNAs are detected in all follicular cell types with varying expression level. MicroRNAs like miR-640, miR-526b* and miR-381 were abundant at higher level in theca cells, while miR-373, miR-30e* and miR-19b-1* expressed more in COCs. However, no significant differences were observed across different cell types in the expression of miR-654-5p and miR-29c. Although the origin of extra-cellular miRNAs in body fluids remains elusive, several reports demonstrated that the origin of extra-cellular miRNAs is closely related to the surrounding cells. While a common set of miRNAs were found in equine follicular fluid and surrounding follicular cells [20], blood cells are reported to be the major contributor of circulating miRNAs in serum [55]. Therefore, the presence of significant level of candidate extra-cellular miRNAs in the surrounding follicular cells could enable us to postulate that the majority of extra-cellular miRNAs in follicular fluid originated from various cell types in the course of their communication during oocyte growth.

In order to elucidate the possible exchange of molecular signals including miRNAs in form of exosome between follicular cells we have investigated the ability of granulosa cells to take up exosomes isolated from follicular fluid. Prior to uptake experiment, the stability of exosome coupled miRNA under *in vitro* culture environment was determined and result showed that exosome coupled miRNAs were stable not only up to 24 hours (**Figure 5**) but even until 48 (data not shown). Similar studies have also shown the stability of circulating serum miRNAs [40], breast milk exosomal miRNA at room temperature and multiple freeze-thaw cycle [56]. This may provide a great potential for functional analysis of exosome mediated transport of molecules for various cellular processes under *in vitro* conditions. Following the confirmation of exosomal miRNA stability under culture conditions, we performed exosomes uptake experiment by primary granulosa cells. Fresh exosomes isolated from follicular fluid and labeled by PKH67 fluorescent dye were co-cultured with bovine primary granulosa cells *in vitro* for 24 hrs. The fluorescent microscopy observation of labeled exosomes co-cultured granulosa cells revealed the presence of green fluorescent exosomes in cultured primary granulosa cells. Similar observation was reported in equine granulosa cells [20], microglial cell line [57], immune cells [16] and other cell lines [17]. Fluorescent microscopy result also shows that the majority of the exosomes taken up by granulosa cells were gathered around perinuclear region. Importantly, there were no fluorescent signals in plasma membrane indicating that exosomes were internalized in to granulosa cells *via* endocytosis. If fusion was the dominant pathway for exosomes uptake, plasma membrane would contain fluorescent signal after co-culture of cells and labeled exosomes. Similar results were observed in exosomes internalization by resting PC12 cells [58]. The consequence of transfection of exosome-coupled miRNAs in endogenous miRNA abundance in granulosa cells was investigated by expression analysis of candidate miRNAs in exosomes derived from follicular fluid of either BCB+ or BCB- oocyte origin. Results have evidenced increased level of endogenous miRNA in exosome transfected granulosa cells compared to the control ones. This could enable us to conclude that exosome mediated exchange of miRNAs in follicular microenvironment is an important way of communication between follicular cells. However, the transfer of other miRNAs through these exosomes and their enrichment in granulosa cells and their subsequent contribution to the regulation of the same target genes or other genes cannot be ruled out. In addition to the complexity of miRNA functional studies, this fact will be a challenge for future studies.

The presence of an extra-cellular miRNA mediated functional changes at gene level in surrounding follicular cells was elucidated by studying the relative mRNA abundance of selected transcripts in exosome co-cultured granulosa cells. Subsequently, we found that transfection of exosomes coupled miRNAs to primary granulosa cells resulted in significant changes in the expression of 7 selected target genes. As shown in **Figure 9**, six genes are down-regulated due to exosome transfection in granulosa cells compared to the untreated control which is associated with elevated level of endogenous targeting miRNAs as shown in **Figure 8**. These down-regulated target genes are known to be involved in different developmental process and cellular network [59], [60], [61], [62], [63], [64]. However, the VEGFA gene, which is known to play a role in improving neovascularization and vascular permeability close to the developing of goat preantral follicles [65] has shown increased abundance after exosome mediated miRNA transfection of granulosa cells. Taken together, results of the present study demonstrate the potential of extra-cellular miRNAs in follicular fluid in regulation of transcripts, which are involved in various processes associated with follicular development, in surrounding follicular cells. However, further investigations are needed to prove these phenomena in bovine granulosa cells.

In conclusion, the present study confirmed the presence of extra-cellular miRNAs in bovine follicular fluid and also demonstrated the exosome and non-exosome mediated transport of miRNAs in follicular microenvironment. In addition, extracellular miRNAs are also bound to Ago2 protein (a part of the RNA-induced silencing complex) in follicular fluid. The stability of exosome-coupled miRNA under *in vitro* culture condition will pave the way for functional analysis of cell to cell communication under *in vitro* environment. Finally, by the comparison of the miRNA expression profiles in follicular fluid of growing and fully grown oocyte source, we have identified several extra-cellular miRNAs which may be associated with growth status of oocyte. However, further functional investigation based on this data could help to find out key regulatory extra-cellular miRNAs controlling oocyte developmental competence by facilitating cell-to-cell communication in follicular environment.

## Supporting Information

Figure S1 **Venn diagram showing the number of detected miRNAs in exosomal and non-exosomal fraction of follicular fluid.** From a total of 748 miRNAs used in the PCR panel 509 and 356 miRNAs were detected (with threshold cycle value of ≤35 in real time PCR analysis) in exosomal and non-exosomal fraction of bovine follicular fluid respectively.
(TIF)

Table S1 List of miRNAs detected^Δ^ in follicular fluid derived from follicles containing a growing vs fully grown oocyte.
(DOC)

Table S2 List of differentially expressed microRNAs and top 6 predicted^Δ^ target genes.
(DOC)

Table S3 Gene ontology analysis* potential target genes of miRNAs differentially expressed in exosomal and non-exosomal fraction of follicular fluid derived from follicle containing growing vs. fully grown oocyte.
(DOC)

Table S4 The list of enriched pathways^Δ^ (P<0.01) by the genes predicted to be targeted by differentially expressed miRNAs in exosomal fraction of follicular fluid derived from follicle containing growing vs. fully grown oocytes.
(DOC)

Table S5 The list of enriched pathways^Δ^ (P<0.01) by the genes predicted to be targeted by differentially expressed miRNAs in follicular fluid from follicles containing growing vs fully grown oocytes.
(DOC)

Table S6 Details of primers used for quantitative real-time PCR analysis of selected target.
(DOC)
