# Supplementary figures and images for: Exosomal and Non-Exosomal Transport of Extra-Cellular microRNAs in Follicular Fluid: Implications for Bovine Oocyte Developmental Competence
Source: PLoS One. 2013 Nov 4;8(11):e78505. doi: 10.1371/journal.pone.0078505 (PMC3817212; doi:10.1371/journal.pone.0078505)

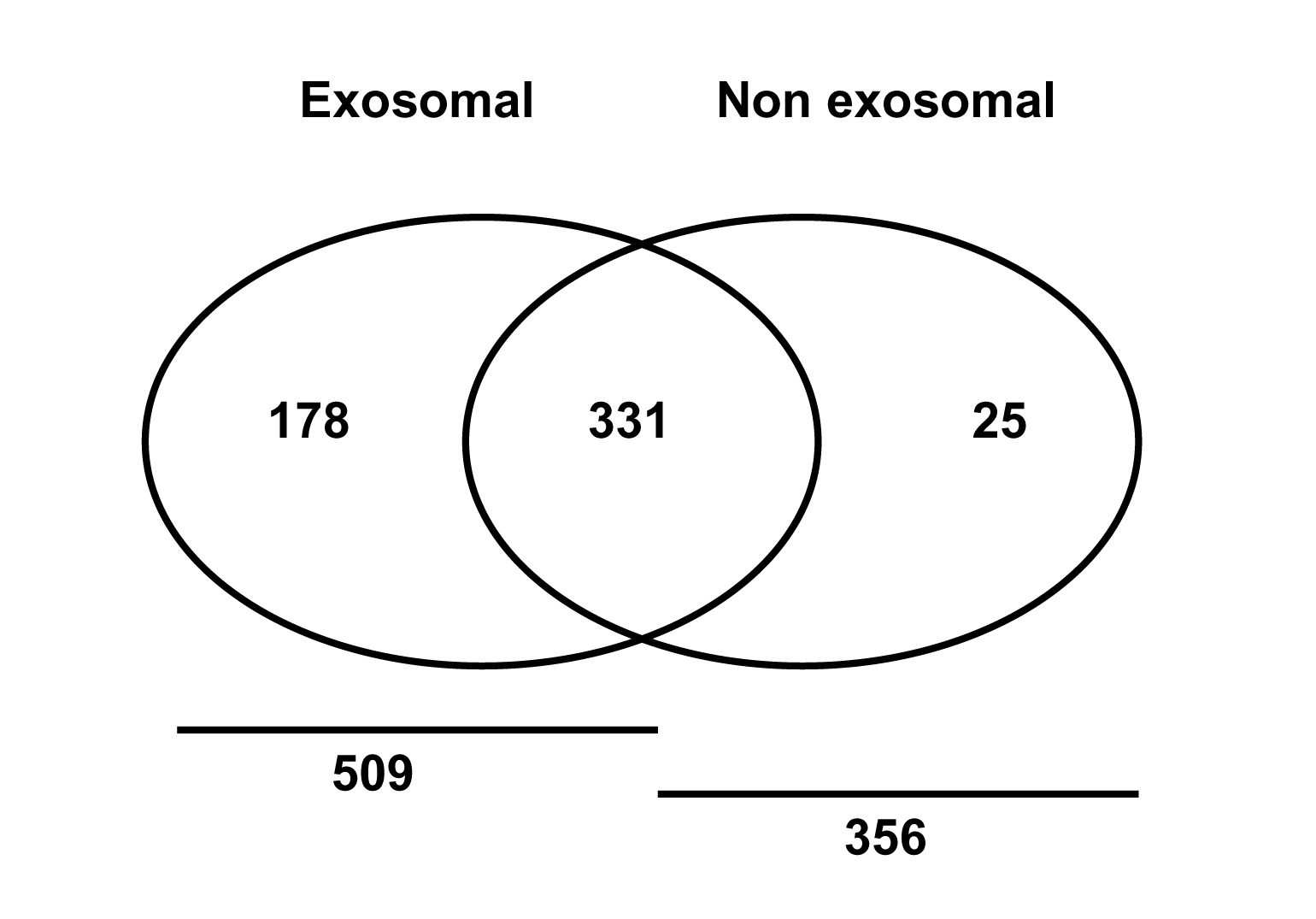

Supplement: Figure S1 — Venn diagram showing the number of detected miRNAs in exosomal and non-exosomal fraction of follicular fluid. From a total of 748 miRNAs used in the PCR panel 509 and 356 miRNAs were detected (with threshold cycle value of ≤35 in real time PCR analysis) in exosomal and non-exosomal fraction of bovine follicular fluid respectively. (TIF) [file pone.0078505.s001.tif]
