# Supplementary material for: Exosomal and Non-Exosomal Transport of Extra-Cellular microRNAs in Follicular Fluid: Implications for Bovine Oocyte Developmental Competence
Source: PLoS One. 2013 Nov 4;8(11):e78505. doi: 10.1371/journal.pone.0078505 (PMC3817212; doi:10.1371/journal.pone.0078505)
Supplement: Table S1 — List of miRNAs detectedΔ in follicular fluid derived from follicles containing a growing vs fully grown oocyte. (DOC) [file pone.0078505.s002.doc]

| **Detected only in Exosomes** | **Detected in both Exosomal and non exosomal portion** | **Detected only in non exosomal structures** |
| --- | --- | --- |
| hsa-let-7c*, hsa-let-7f-2*, hsa-let-7g*, hsa-let-7i, miR-105, miR-10a, miR-1183, miR-1185, miR-122, miR-1227, miR-1247, miR-1249, miR-1256, miR-1272, miR-127-3p, miR-127-5p, miR-128, miR-129*, miR-129-5p, miR-130a*, miR-132*, miR-135a*, miR-135b, miR-136, miR-137, miR-143*, miR-146a, miR-147, miR-148b*, miR-153, miR-155*, miR-15a*, miR-17*, miR-181c, miR-181c*, miR-181d, miR-182*, miR-187, miR-188-3p, miR-190, miR-190b, miR-1911*, miR-192*, miR-193b*, miR-196a, miR-196b, miR-196b*, miR-198, miR-200a, miR-200c, miR-200c*, miR-205, miR-206, miR-208b, miR-20a*, miR-20b*, miR-21*, miR-2113, miR-214*, miR-216b, miR-22*, miR-223*, miR-23a*, miR-23b*, iR-24-1*, miR-24-2*, miR-26a-1*, miR-27b*, miR-28-3p, miR-296-3p, miR-296-5p, miR-297, miR-299-3p, miR-299-5p, **miR-29a***, miR-29c*, miR-301b, miR-31, miR-323-3p, miR-326, **miR-33a***, miR-335*, miR-338-3p, miR-338-5p, miR-33a*, miR-33b, miR-340, miR-342-5p, miR-345, miR-34a, miR-34a*, miR-34b, miR-361-3p, miR-362-3p, miR-365*, miR-370, miR-371-5p, miR-372, miR-377, miR-378, miR-379*, miR-383, miR-384, miR-409-5p, miR-410, miR-411, miR-411*, miR-429, miR-431*, miR-450b-5p, **miR-451**, miR-454, miR-487a, miR-487b, miR-490-5p, miR-492, miR-493*, miR-494, miR-502-3p, miR-504, miR-514, miR-517a, miR-518f, miR-520a-5p, miR-520d-3p, miR-520d-5p, miR-520h, miR-524-3p, miR-525-3p, miR-526a, miR-541, miR-544, miR-548c-3p, miR-551b*, miR-556-5p, miR-566, miR-590-5p, miR-596, miR-600, miR-601, miR-605, **miR-608**, miR-615-5p, miR-616, miR-620, miR-627, miR-629*, miR-631, miR-649, miR-652, miR-660, miR-663b, miR-671-3p, miR-671-5p, miR-675*, miR-708, miR-7-1*, miR-767-5p, miR-769-5p, miR-876-5p, miR-877*, miR-885-5p, miR-886-3p, miR-888*, miR-891a, miR-9, miR-9*, miR-924, miR-92b*, miR-93*, miR-937, miR-941, miR-942, miR-95, miRPlus-C1076, miRPlus-C1089, miRPlus-D1061 | hsa-let-7a, hsa-let-7a*, hsa-let-7b, hsa-let-7b*, **hsa-let-7c**, hsa-let-7d, hsa-let-7d, hsa-let-7d*, has, let-7e, hsa-let-7f, hsa-let-7f-1*, hsa-let-7g, **hsa-let-7i***, miR-1, miR-100, miR-101, **miR-103**, miR-103-2*, miR-103-as, miR-105*, miR-106a, miR-106b, miR-106b*, miR-107, miR-10a*, miR-10b, miR-10b*, miR-1179, miR-1181, miR-1207-5p, miR-1224-3p, miR-1237, miR-124, miR-1254, miR-125a-3p, miR-125a-5p, miR-125b, miR-125b-1*, miR-125b-2*, miR-126, miR-126*, miR-1260, miR-1266, miR-1272, miR-129-3p, miR-1296, miR-130a, miR-130b, miR-130b*, **miR-132**, **miR-133a**, miR-133b, miR-134, miR-135a, miR-138, miR-138-2*, miR-139-5p, miR-140-3p, miR-140-5p, miR-141, miR-141*, miR-142-3p, miR-142-5p, miR-143, miR-145, miR-1468, miR-146b-5p, miR-1471, miR-147b, miR-148a, miR-148a*, miR-148b, miR-149, miR-149*, miR-150, miR-151-3p, miR-151-5p, miR-152, miR-1538, miR-1539, **miR-155**, **miR-15a**, miR-15b, miR-15b*, miR-16. miR-17, miR-181a, **miR-181a***, miR-181a-2*, miR-181b, miR-183, **miR-184**, miR-185, **miR-186**, miR-187*, miR-188-5p, miR-18a, miR-18a*, miR-18b, miR-1909, miR-191, **miR-191***, miR-1913, miR-1914, miR-192, **miR-193a-3p**, miR-193a-5p, miR-193b, miR-194, miR-194*, miR-195, miR-195*, miR-197, miR-1972, miR-1979, miR-199a-3p, **miR-199a-5p**, miR-199b-5p, miR-19a, miR-19b, **miR-19b-1***, miR-200b, miR-200b*, miR-202, miR-202*, miR-203, miR-204, miR-20a, **miR-21**, mir-210,, miR-2110, miR-212, miR-214, miR-215, miR-218, miR-218-1*, miR-219-5p, miR-22, **miR-221**, miR-221*, **miR-222**, miR-224, miR-224*, miR-23a, miR-23b, miR-24, miR-25, miR-25*, miR-26a, miR-26a-2*, miR-26b, **miR-26b***, miR-27a, miR-27b, miR-28-5p, **miR-29a**, miR-29b, miR-29b-1*, **miR-29c**, miR-300, miR-301a, **miR-302c**, miR-302d*, miR-30a, miR-30a*, **miR-30b**, miR-30b*, miR-30c, miR-30c-1*, miR-30c-2*, miR-30d, miR-30d*, miR-30e, **miR-30e***, miR-31*, miR-320a, miR-320b, **miR-324-3p**, miR-324-5p, **miR-328**, miR-329, miR-330-3p, miR-330-5p, miR-331-3p, miR-331-5p, miR-335, miR-339-5p, miR-33a, miR-33b*, miR-342-3p, miR-346, miR-34c-3p, miR-34c-5p, miR-361-5p, miR-362-5p, **miR-363**, **miR-365**, miR-369-5p, **miR-373**, miR-373*, miR-374a, miR-374b, miR-375, miR-376a, miR-376a*, miR-376b, miR-376c, miR-378*, miR-379, **miR-381**, miR-382, miR-409-3p, miR-421, miR-422a, miR-423-3p, **miR-423-5p**, miR-423-5p, miR-424, **miR-424***, miR-425, **miR-425***, miR-432, miR-433, miR-449a, **miR-449b**, miR-450am, **miR-450b-3p**, miR-452, miR-454*, miR-455-3p, miR-455-5p, miR-483-3p, miR-484, miR-485-3p, miR-486-3p, miR-486-5p, miR-490-3p, miR-491-3p, **miR-491-5p**, miR-493, miR-495, miR-496, miR-497, miR-499-5p, miR-500a, miR-502-5p, miR-503, **miR-505**, miR-505*, miR-509-3p, miR-510, miR-518c, miR-518f*, **miR-519d**, miR-521, miR-526b, **miR-526b***, miR-532-3p, miR-532-5p, miR-542-5p, miR-543, miR-545, miR-550a*, miR-551b, miR-552, miR-555, miR-558, miR-562, miR-564, miR-571, **miR-573**, miR-574-3p, miR-577, **miR-582-5p**, miR-589, miR-592, miR-593, miR-595, miR-598, miR-615-3p, **miR-617**, miR-624*, miR-628-3p, miR-628-5p, miR-638, miR-639, **miR-640**, miR-643, **miR-654-5p**, **miR-659**, miR-661, miR-662, miR-663, miR-664, miR-665, miR-668, miR-675b, miR-7, miR-708*, miR-720, miR-744, miR-744*, miR-758, miR-760, miR-765, miR-766, **miR-873**, miR-876-3p, miR-877, miR-885-3p, miR-886-5p, miR-888, miR-92a, miR-92a-1*, miR-92b, miR-93, miR-933, **miR-934**, miR-936, miR-940, miR-943, miR-98, miR-99a, **miR-99a***, miR-99b, miR-99b*, miRPlus-A1027, miRPlus-A1031, miRPlus-C1066 | **hsa-let-7a-2***, **miR-101***, miR-1244, miR-1267, **miR-145***, miR-185*, miR-216a, **miR-223**, miR-302a, miR-367, miR-431, miR-498, miR-501-5p, miR-518b, miR-518c*, miR-548j, miR-549, miR-572, miR-576-5p, miR-584, miR-622, miR-629, miR-646, **miR-654-3p**, miR-802, miR-887 |
| ^Δ^miRNAs were considered as detected only when the threshold cycle (Ct) value is less than 35.  Differentially expressed miRNAs (P<0.05 & 2 fold change) are bold and underlined | | |

**Table S1.** List of miRNAs detected^Δ^ in follicular fluid derived from follicles containing a growing vs fully grown oocyte
