# Supplementary material for: Exosomal and Non-Exosomal Transport of Extra-Cellular microRNAs in Follicular Fluid: Implications for Bovine Oocyte Developmental Competence
Source: PLoS One. 2013 Nov 4;8(11):e78505. doi: 10.1371/journal.pone.0078505 (PMC3817212; doi:10.1371/journal.pone.0078505)
Supplement: Table S2 — List of differentially expressed microRNAs and top 6 predictedΔ target genes. (DOC) [file pone.0078505.s003.doc]

**Table S2**. List of differentially expressed microRNAs and top 6 predicted^Δ^ target genes

|  | | | |
| --- | --- | --- | --- |
| **Exosomal Portion (Growing vs. fully grown)** | | **Non Exosomal Portion (Growing vs. fully grown)** | |
| **MicroRNA Name** | **Gene symbol** | **MicroRNA**  **Name** | **Gene symbol** |
| miR-654-5p | HPS1, VDR, GLE1, GLE1, MGLL, ZCCHC16 | miR-19b-1* | ZNF117, EDNRB, PGR, CGGBP1, C6orf168, EIF2S1 |
| miR-640 | C4orf15, AFF2, RPS23, MYO6, MYO1B, ZFP91 | miR-29c | COL3A1, COL1A2, COL4A2, TDG, COL1A1, CAMK2G |
| miR-582-5p | ITGA6, RB1, COL5A2, THRB, TYRP1, CD3E | miR-659 | TMEM156, VEZT, ZNF295, FAM107B, ZNF280C, ACVR1C |
| miR-449b | VAT1, LGR4, SVOP, FOXP1, ANK3, NAV1 | miR-29a | DNMT3A, RLF, PPP1R13B, TUBB2A, RNF19A, IREB2 |
| miR-155 | BACH1, ZIC3, UBQLN1, SATB1, KRAS, DYNC1I1 | miR-424* | HNRNPA0, METAP2, LRP1, SMC2, SRSF12, FOS |
| miR-573 | ESR1, MUT, PTS, TSHR, KCNJ2, FLJ42177 | miR-133a | CDC2L5, SGK1, SGPP1, SGMS2, SEC61B, ELF2 |
| miR-451 | ZNF644, MEX3C, C11orf30, GADL1, CXCL16, CTNNBIP1 | miR-193a-3p | E2F6, PPP2R5C, EN2, WDR68, MCL1, SLC39A5 |
| miR-221 | CDKN1B, FOS, ASB7, C12orf30, PPARGC1A, INSIG1 | miR-617 | RBM33, FLJ31818, SORBS1, TMEM19, ALDH1A2, MTPN |
| miR-363 | RGS17, REV3L, TBL1XR1, LOC91461, MAN2A1, FBXW7 | miR-145* | PCDH15, TDRD6, CCDC88A, SLC30A5, PRPF4B, PBX3 |
| miR-199a-5p | CCNL1, PAN3, AKAP1, STK4, ZNF512B, SLC24A3 | miR-423-5p | SCAF1, C20orf54, ADAM19, PML, WNT3A, ATCAY |
| hsa-let-7c | HMGA2, TRIM71, MYC, CCNJ, RSPO2, BIN3 | miR-365 | CSK, YTHDF2, ANKRD11, LAMP2, SOCS5, WDR37 |
| miR-491-5p | DNAJB5, ZNF644, AMD1, APPBP2, NFIB, MYST2 | miR-99a* | TMEM87A, SOCS4, RNF10, THUMPD1, NRK, NUP160 |
| miR-21 | RP2, YAP1, ADNP, ARHGEF7, C17orf39, BAHD1 | miR-505 | DHX15, SFRS2IP, MYBL1, KIAA0408, FAM122A, C12orf23 |
| miR-132 | SIRT1, TIMM9, C5orf13, PRICKLE2, DCUN1D3, NMNAT2 | miR-101* | RAP1A, SGCB, QKI, SNX10, PBK, CHML |
| miR-873 | SLC46A1, FBXO17, SLC35E1, PRKRIP1, C22orf29, SH3TC2 | miR-15a | BCL2, ADRB2, GHR, PTCH1, CASR, PAFAH1B1 |
| miR-324-3p | ATRX, GPATCH8, KIAA0265, ABCA1, LPHN1, GIT1 | miR-222 | CDKN1B, ARID1A, VAPB, POGZ, CDC2L6, TMCC1 |
| miR-450b-3p | PPP1R16B, DCUN1D1, CRAMP1L, SLC25A23, C20orf121, LPAL2 | miR-103 | KIF5A, EPHA7, LATS2, MARCH3, RUNX1T1, LPHN3 |
| miR-191* | PHTF2, DCAF12L1, TSN, ANK1, PPP4R1, DLGAP2 | miR-654-3p | TNFSF11, C5orf23, CEP97, RNF128, FUNDC2, ARID4A |
| miR-26b* | MAP3K13, XPR1, SPOPL, ANTXR1, ZNF397, MOSPD1 | miR-532-5p | NAT2, COL4A4, KCNE1, MYO5A, MYOC, NCF2 |
| miR-1272 | PSEN1, AGA, AGL, FAS, ATM, ATP7A, ATP7B | miR-145 | SEMA3A, AKAP12, ACTG1, CLINT1, ADPGK, PAPD4 |
| miR-29a* | ZFX, GPR180, GPR180, FAM49B, SC5DL, C11orf41 | miR-574-3p | TMCC1, CLTC, FBXL5, BACE1, MESDC1, ERC2 |
| miR-30b | GLDC, ITGA6, SGCB, SOX9, IL1A, PPP3R1 | miR-184 | EPB41L5, NUS1, SF1, EIF2C2, FZD1, PRKCB1 |
| miR-33a* | FMR1, C1orf27, YAF2, PHC3, C4orf40, SH3TC2 | miR-425-3p | OTX1, GOLGA7B, FAM175A, EPB41L5, TLE3, KIFC2 |
| miR-526b* | MAP3K2, FGD4, FYCO1, APBB2, ADARB1, RRAGD | miR-186 | JAG1, MITF, OSBPL8, CCNT2, EIF4E, PPP1CC |
| miR-373 | TFAP4, LATS2, ARHGEF3, TNFAIP1, CFL2, AOF1 | miR-519d | NCOA3, BRMS1L, BTBD10, PKNOX1, RPS6KA5, ZFYVE9 |
|  |  | miR-302c | ZNFX1, MEF2C, LUC7L2, TOX, DERL2, DMTF1 |
|  |  | miR-934 | ZNF215, PSIP1, SPCS3, FBXL17, ZNF557, ZDHHC11 |
|  |  | miR-30e* | ARMC1, LGI2, TNRC6B, AAK1, NUFIP2, CREB1 |
|  |  | miR-18a* | LONRF2, CSNK1D, PDP1, PPFIA4, CAMKV, MSRB3 |
|  |  | miR-381 | RHOQ, SEMA6D, ANKRD50, CLCF1, EIF4G2, BRD7 |
| ^Δ^Genes predicted by miRecord or miRBD | | | |
