# Supplementary material for: Exosomal and Non-Exosomal Transport of Extra-Cellular microRNAs in Follicular Fluid: Implications for Bovine Oocyte Developmental Competence
Source: PLoS One. 2013 Nov 4;8(11):e78505. doi: 10.1371/journal.pone.0078505 (PMC3817212; doi:10.1371/journal.pone.0078505)
Supplement: Table S3 — Gene ontology analysis* potential target genes of miRNAs differentially expressed in exosomal and non-exosomal fraction of follicular fluid derived from follicle containing growing vs. fully grown oocyte. (DOC) [file pone.0078505.s004.doc]

| **Exosomal portion** | | **Non exosomal portion** | | |
| --- | --- | --- | --- | --- |
| **GO ID & terms** | **Enrichment**  **P value** | | **GO ID & terms** | **Enrichment**  **P value** |
| GO:0006350~transcription | 9.69E-32 | | GO:0045449~regulation of transcription | 4.16E-14 |
| GO:0045449~regulation of transcription | 3.18E-29 | | GO:0015031~protein transport | 1.05E-11 |
| GO:0051252~regulation of RNA metabolic process | 1.10E-20 | | GO:0045184~establishment of protein localization | 1.33E-11 |
| GO:0006355~regulation of transcription, DNA-dependent | 2.20E-20 | | GO:0051173~positive regulation of nitrogen compound metabolic process | 1.61E-11 |
| GO:0007242~intracellular signaling cascade | 1.19E-18 | | GO:0010628~positive regulation of gene expression | 5.45E-11 |
| GO:0006814~sodium ion transport | 1.50E-17 | | GO:0045941~positive regulation of transcription | 1.74E-10 |
| GO:0055085~transmembrane transport | 3.42E-15 | | GO:0031328~positive regulation of cellular biosynthetic process | 1.96E-10 |
| GO:0007264~small GTPase mediated signal transduction | 6.46E-14 | | GO:0009891~positive regulation of biosynthetic process | 3.32E-10 |
| GO:0015837~amine transport | 3.32E-13 | | GO:0051252~regulation of RNA metabolic process | 4.77E-10 |
| GO:0015031~protein transport | 3.37E-13 | | GO:0007167~enzyme linked receptor protein signaling pathway | 6.00E-10 |
| GO:0006865~amino acid transport | 8.93E-13 | | GO:0006350~transcription | 9.70E-10 |
| GO:0046942~carboxylic acid transport | 5.58E-12 | | GO:0051254~positive regulation of RNA metabolic process | 1.21E-09 |
| GO:0019941~modification-dependent protein catabolic process | 6.45E-12 | | GO:0008104~protein localization | 1.49E-09 |
| GO:0015849~organic acid transport | 7.81E-12 | | GO:0045893~positive regulation of transcription, DNA-dependent | 2.80E-09 |
| GO:0046034~ATP metabolic process | 1.39E-11 | | GO:0046907~intracellular transport | 4.31E-09 |
| Note: only top 15 terms from each portion are listed here. | | | | |

**Table S3.** Gene ontology analysis potential target genes of miRNAs differentially expressed in exosomal and non exosomal portion of follicular fluid derived from follicle containing growing vs. fully grown oocyte.
