# Supplementary material for: Exosomal and Non-Exosomal Transport of Extra-Cellular microRNAs in Follicular Fluid: Implications for Bovine Oocyte Developmental Competence
Source: PLoS One. 2013 Nov 4;8(11):e78505. doi: 10.1371/journal.pone.0078505 (PMC3817212; doi:10.1371/journal.pone.0078505)
Supplement: Table S4 — The list of enriched pathwaysΔ (P<0.01) by the genes predicted to be targeted by differentially expressed miRNAs in exosomal fraction of follicular fluid derived from follicle containing growing vs. fully grown oocytes. (DOC) [file pone.0078505.s005.doc]

**Table S4.** The list of enriched pathways^Δ^ (P<0.01) by the genes predicted to be targeted by differentially expressed miRNAs in exosomal portion of follicular fluid derived from follicle containing growing vs. fully grown oocytes.

| **KGG Pathway Term** | **Count** | **P value** | **Gene Symbol^ΔΔ^** |
| --- | --- | --- | --- |
| **Exosomal portion** |  |  |  |
| hsa04120:Ubiquitin mediated proteolysis | 46 | 6.54E-18 | UBE2Z, SYVN1, UBE3B, XIAP, UBE3A, UBE2G1, UBE2G2, UBA6, UBE3C, STUB1, UBE2R2, UBE2D4, UBE2D3, UBE2D2, WWP2, WWP1, UBE2D1, TRIP12, UBE4A, VHL, SOCS3, SOCS1, UBE2J1, UBE2F, UBE2I, UBE2NL, UBE2J2, UBOX5, UBE2L3, UBE2Q2, UBE2B, UBE2N, TRIM37, UBE2O, UBE2E3, UBA1, UBE2K, TRIM32, UBE2M, TCEB2, UBE2W, SIAH1, SMURF2, TCEB1, SMURF1, UBE2E1 |
| hsa04722:Neurotrophin signaling pathway | 48 | 3.35E-09 | NFKB1, MAPKAPK2, IRAK4, IRAK3, MAP3K5, KRAS, MAP3K3, RAC1, RHOA, PIK3CA, PIK3R5, MAP2K7, RAPGEF1, PIK3R1, PIK3R2, MAP2K5, PDK1, IRAK2, IRS2, NTF3, MAP2K2, PIK3CD, MAPK11, MAPK10, KIDINS220, IRS1, PTPN11, RPS6KA5, NTRK3, MAPK1, NRAS, RPS6KA3, RPS6KA4, PRDM4, RPS6KA1, PLCG1, PSEN1, RPS6KA2, MAPK14, NTRK2, RAP1A, MAPK9, MAPK8, RAP1B, MAPK7, NGFR, IKBKB, NGF |
| hsa04010:MAPK signaling pathway | 79 | 3.51E-08 | MEF2C, PDGFB, NFKB1, NFKB2, PRKX, MAP3K7, MAX, MAP3K5, PAK2, MAPT, RRAS, PRKACA, PAK1, PRKACB, MAP2K7, MYC, IL1A, MAP2K6, MAP2K5, PRKCA, MAP4K3, MAPK1, MAP4K4, PDGFRA, MAPK9, HSPB1, PLA2G6, PDGFRB, MAPK8, MAPK7, PLA2G3, MAP3K14, MAP3K13, PLA2G5, MAP3K12, PLA2G2F, NGF, MAP3K11, IL1R1, MRAS, MAP4K2, MKNK2, PPM1A, PPP3R1, MAPKAPK3, PPP3R2, MAPKAPK2, PPM1B, HSPA1B, KRAS, MAP3K3, RAC2, MAP3K2, RASGRP1, RAC1, PPP3CB, PPP3CC, NFATC4, PPP3CA, RASA1, NTF3, MAP2K2, NLK, MAP2K3, NF1, MAP2K4, MAPK11, MAPK10, RPS6KA5, NRAS, RPS6KA3, RPS6KA4, RPS6KA1, RPS6KA2, MAPK14, NTRK2, RAP1A, RAP1B, IKBKB |
| hsa04370:VEGF signaling pathway | 33 | 4.36E-08 | PTGS2, PPP3R1, MAPKAPK3, PPP3R2, MAPKAPK2, PXN, KRAS, RAC2, RAC1, NFAT5, PPP3CB, PPP3CC, PIK3CA, NFATC4, PIK3R5, PPP3CA, PIK3R1, NFATC1, PIK3R2, PRKCA, MAP2K2, PIK3CD, MAPK11, KDR, MAPK1, NRAS, PLCG1, MAPK14, PLA2G6, HSPB1, PLA2G3, PLA2G5, PLA2G2F |
| hsa04130:SNARE interactions in vesicular transport | 26 | 5.87E-08 | SNAP29, STX5, STX4, STX1A, STX3, STX2, VTI1A, STX17, STX16, VAMP4, VAMP3, SNAP23, VAMP2, STX11, VAMP1, YKT6,NRAS, KRAS, PIK3CA, PIK3R5, RARA, RUNX1, IKBKB, MYC, PIK3R1, PIK3R2 |
| hsa04910:Insulin signaling pathway | 48 | 7.56E-08 | PRKAG3, PHKA1, MKNK2, HK2, RHOQ, RPS6KB1, PRKX, PRKAR2B, PPP1R3D, PDPK1, PRKAR2A, PPP1R3C, KRAS, PPP1R3B, PIK3CA, GYS2, PRKAA1, PIK3R5, PRKACA, INPP5D, PRKACB, RAPGEF1, INSR, PIK3R1, PIK3R2, IRS2, PTPRF, MAP2K2, PIK3CD, PRKAB2, PRKCI, IGF2, MAPK10, PPP1R3A, PPP1CC, IRS1, PPARGC1A, PPP1CB, PCK1, MAPK1, NRAS, PRKAR1B, PRKAR1A, MAPK9, RHEB, MAPK8, PTPN1, IKBKB |
| hsa04510:Focal adhesion | 61 | 6.51E-07 | PDGFB, PGF, PIP5K1C, PTEN, PAK6, PAK7, PDPK1, PAK2, PAK4, RHOA, PIK3CA, PDGFC, PDGFD, PAK1, RAPGEF1, PRKCA, ROCK1, MYLK3, PIK3CD, MYLK2, PPP1CC, PPP1CB, MAPK1, LAMC3, PDGFRA, PDGFRB, MAPK9, RELN, MAPK8, LAMC1, PARVA, ITGB4, ITGA11, ITGA10, ITGB3, PXN, IGF1R, LAMB3, RAC2, ITGB8, ITGAV, RAC1, PPP1R12A, PIK3R5, PIK3R1, PIK3R2, MET, IGF1, ITGA2, ITGA3, MAPK10, HGF, KDR, ITGA9, LAMA3, ITGA6, ITGA5, LAMA5, RAP1A, RAP1B, MYLK |
| hsa04810:Regulation of actin cytoskeleton | 63 | 1.57E-06 | PDGFB, PIP5K1B, IQGAP3, PIP5K1C, IQGAP2, PIP5K1A, PAK6, PAK7, PAK2, PAK4, RHOA, PIK3CA, RRAS, PDGFC, PDGFD, PAK1, MSN, ROCK1, LIMK1, MYLK3, PIK3CD, MYLK2, MYH9, PPP1CC, PPP1CB, MAPK1, PDGFRA, PDGFRB, ITGAL, MRAS, ITGB4, ITGA11, ITGA10, RDX, ITGB3, PXN, PFN2, KRAS, RAC2, ITGAX, ITGB8, ITGAV, RAC1, PPP1R12A, PIK3R5, PIK3R1, PIK3R2, MAP2K2, ITGA2, NCKAP1L, IGF2, ITGA3, NCKAP1, NRAS, ITGA9, ITGA6, ITGA5, MYH14, PIP4K2A, PIP4K2C, MYLK, PIP4K2B, MYH10 |
| hsa04062:Chemokine signaling pathway | 59 | 3.34E-06 | ADCY3, ADCY1, ADCY2, ADCY7, ADCY6, FOXO3, CXCL12, CXCL10, CXCR5, CXCR6, GNG2, GNG3, GNG4, GNG5, CHUK, AKT3, GNG7, AKT2, CCNL2, CCR9, CCR8, CCR7, CCR6, CRKL, GNB1, CCR2, CX3CR1, GNB5, GNB4, GNB3, CXCL1, CCL1, GNAI3, GNAI2, GNAI1, GRB2, CXCL9, ADRBK2, ADRBK |
| hsa04350:TGF-beta signaling pathway | 20 | 3.08E-05 | SMAD9, SMAD7, SMAD6, TGFBR1, SMAD5, TGFBR2, TGFB3, SMAD4, SMAD3, SMAD2, SMAD1, TGFB1, SP1, ZFYVE16, SMURF2, SMURF1, THBS1, THBS2, THBS3, TFDP1 |
| ^Δ^Only top 8 KEGG pathways according to P value are listed here.  ^ΔΔ^The gene symbol is presented as in NCBI at http://www.ncbi.nml.nih.gov | | | |
