# Supplementary material for: Exosomal and Non-Exosomal Transport of Extra-Cellular microRNAs in Follicular Fluid: Implications for Bovine Oocyte Developmental Competence
Source: PLoS One. 2013 Nov 4;8(11):e78505. doi: 10.1371/journal.pone.0078505 (PMC3817212; doi:10.1371/journal.pone.0078505)
Supplement: Table S5 — The list of enriched pathwaysΔ (P<0.01) by the genes predicted to be targeted by differentially expressed miRNAs in follicular fluid from follicles containing growing vs fully grown oocytes. (DOC) [file pone.0078505.s006.doc]

| **KGG Pathway Term** | **Count** | **P value** | **Gene Symbol^ΔΔΔ^** |
| --- | --- | --- | --- |
| hsa04310:Wnt signaling pathway | 39 | 7.78E-09 | WNT5A, WNT16, PPARD, WNT5B, PPP2R5D, WNT3A, PPP2R5C, PPP3R1, PPP3R2, PRKX, WNT2, PPP2CA, PPP2CB, RAC1, PPP3CB, PRKACA, PPP3CA, PRKACB, PLCB1, PRKCA, PPP2R1A, TBL1XR1, TCF7, ROCK1, ROCK2, VANGL2, SMAD3, SMAD2, SKP1, SENP2, SFRP5, WNT7B, SFRP1, PSEN1, SFRP4, PRICKLE2, SIAH1, TBL1X, WNT7A |
| hsa05200:Pathways in cancer | 39 | 5.90E-08 | E2F2, FGF5, E2F3, FGF7, ARNT2, FOXO1, NFKB1, FGF12, MMP2, GLI3, ACVR1C, CCNE2, CDC42, CUL2, ACVR1B, FOS, MAX, CCNE1, CDKN2A, HHIP, FAS, FGF2, AKT3, AKT2, CTBP1, CTBP2, BCR, CYCS, LEF1, FGF23, CDK6, CTNNA1, CDK2, CTNNA2, MAPK1, CCDC6, CCND1, CRKL, EP300 |
| hsa04510:Focal adhesion | 37 | 5.25E-07 | ACTG1, PAK6, CDC42, PAK7, ARHGAP5, PAK4, PAK1, COL11A1, AKT3, AKT2, ACTN2, FLNB, MAPK1, CCND1, CRKL, CCND3, CCND2, LAMC3, MAPK3, COL1A2, LAMC2, LAMC1, COL1A1, CAV3, CAV2, GRB2, COL3A1, ITGA11, ITGA10, COL2A1, ITGB3, ITGB1, IGF1R, ITGB8, ITGAV, BCL2, COL6A |
| hsa04360:Axon guidance | 31 | 1.91E-06 | PLXNA1, PLXNA2, PPP3R1, PPP3R2, SEMA5A, SEMA5B, PTK2, UNC5B, ROBO1, UNC5A, SEMA3F, RAC1, PPP3CB, SEMA3D, SEMA3C, UNC5D, SEMA3B, PPP3CA, SEMA3A, RASA1, ROCK1, ROCK2, SEMA6A, RND1, SEMA6D, RGS3, SRGAP3, SEMA4C, SEMA4A, SRGAP1, SRGAP2 |
| hsa04130:SNARE interactions in vesicular transpor | 15 | 4.14E-06 | SNAP29, STX5, STX1A, VTI1B, VTI1A, STX12, VAMP8, STX17, VAMP7, STX16, VAMP4, VAMP2, SNAP23, STX11, VAMP1 |
| hsa04512:ECM-receptor interaction | 31 | 8.79E-06 | COL3A1, DAG1, ITGA11, ITGA10, COL2A1, ITGB3, ITGB1, CD47, ITGB8, ITGAV, COL6A3, COL11A1, FN1, COL4A4, COL4A2, COL4A1, HSPG2, ITGA2, ITGA3, ITGA4, COL5A3, COL5A2, COL5A1, LAMA2, ITGA6, LAMC3, ITGA5, COL1A2, LAMC2, COL1A1, LAMC1 |
| hsa05222:Small cell lung cancer | 31 | 8.79E-06 | E2F2, E2F3, NFKB1, BCL2L1, ITGB1, CCNE2, CCNE1, MAX, BCL2, ITGAV, AKT3, AKT2, FN1, COL4A4, COL4A2, COL4A1, CYCS, ITGA2, CDK6, ITGA3, BIRC2, CDK2, LAMA2, CCND1, CDKN1B, ITGA6, LAMC3, LAMC2, APAF1, LAMC1, IKBKB |
| hsa04010:MAPK signaling pathway | 52 | 1.57E-06 | MEF2C, FGF5, FGF7, NFKB1, FGF12, ACVR1C, CDC42, ACVR1B, FOS, MAX, BDNF, MAP3K4, MAPT, PAK1, FAS, MAP2K7, FGF2, IL1A, MAP2K6, AKT3, AKT2, CACNG4, FGF23, FLNB, MAPK1, MAP4K4, CRKL, ARRB2, MAPK3, MAP3K11, FGFR2, FGFR1, IL1R1, GRB2, MRAS, CACNB1, DUSP10, MAPK,MAP2K4, MAPK11, MAPK10, RPS6KA5, NRAS, RPS6KA3, RPS6KA4, RPS6KA1, RPS6KA2, MAPK14, NTRK2, RAP1A, RAP1B, IKBKB |
| ^Δ^Only top 8 KEGG pathways according to P value are listed here.  ^ΔΔ^The gene symbol is presented as in NCBI at http://www.ncbi.nml.nih.gov | | | |

**Table S5.** The list of enriched pathways^Δ^ (P<0.01) by the genes predicted to be targeted by differentially expressed miRNAs in follicular fluid from follicles containing growing vs fully grown oocytes
