# Supplementary material for: Exosomal and Non-Exosomal Transport of Extra-Cellular microRNAs in Follicular Fluid: Implications for Bovine Oocyte Developmental Competence
Source: PLoS One. 2013 Nov 4;8(11):e78505. doi: 10.1371/journal.pone.0078505 (PMC3817212; doi:10.1371/journal.pone.0078505)
Supplement: Table S6 — Details of primers used for quantitative real-time PCR analysis of selected target. (DOC) [file pone.0078505.s007.doc]

**Table S6.** Details of primers used for quantitative real-time PCR analysis of selected target genes.

| **Gene** | **Accession number** | **Primer sequences**  **(5’ -> 3’)** | **Annealing temperature (°C)** | **Product size (bp)** |
| --- | --- | --- | --- | --- |
| GAPDH | NM_001034034 | F:CCAGGGCTGCTTTTAATTCT  R:ATGGCCTTTCCATTGATGAC | 60 | 247 |
| CD44 | NM_174013 | F: CTGAAATGAGGGCCCAGTTA  R: CCAACCCCACTTGAAAGAAA | 57 | 236 |
| BRMS1L | NM_001083428 | F: GCCATCTCTCCAGTTCTGCT  R: AAGTGGGACCCAACTCAGTG | 59 | 211 |
| ZNFX1 | NM_001205716 | F: GTCAGCCAGGAGCGACTTAC  R: GCTGAAGCTCAAACGCTTCT | 58 | 189 |
| ITGA3 | XM_003587418 | F: CAAGTCTGAGGGCCAGAAAC  R: CTCCTTCACCACCAGGAATC | 59 | 265 |
| MAP3K1 | NM_001205906 | F: ACTGGCCAGCATTTCAGTAG  R: TGTGTTTGAGGAGATGCAGA | 56 | 217 |
| SOCS4 | NM_001076218 | F: CATTCTTCAGGGCTTCCATC  R: TGGTTATGACACAGGGCTGA | 57 | 244 |
